# Isolation of endothelial cells, pericytes and astrocytes from mouse brain

**DOI:** 10.1371/journal.pone.0226302

**Published:** 2019-12-18

**Authors:** Florian Bernard-Patrzynski, Marc-André Lécuyer, Ina Puscas, Imane Boukhatem, Marc Charabati, Lyne Bourbonnière, Charles Ramassamy, Grégoire Leclair, Alexandre Prat, V Gaëlle Roullin

**Affiliations:** 1 Faculty of Pharmacy, Université de Montréal, Montreal, Québec, Canada; 2 Department of Neuroscience, Faculty of Medicine, Université de Montréal, Montreal, Québec, Canada; 3 Institute for Multiple Sclerosis Research and Neuroimmunology, University Medical Center Göttingen, Göttingen, Germany; 4 Institut National de la Recherche Scientifique, Armand-Frappier Institute, Laval, Québec, Canada; University of Illinois at Chicago, UNITED STATES

## Abstract

Primary cell isolation from the central nervous system (CNS) has allowed fundamental understanding of blood-brain barrier (BBB) properties. However, poorly described isolation techniques or suboptimal cellular purity has been a weak point of some published scientific articles. Here, we describe in detail how to isolate and enrich, using a common approach, endothelial cells (ECs) from adult mouse brains, as well as pericytes (PCs) and astrocytes (ACs) from newborn mouse brains. Our approach allowed the isolation of these three brain cell types with purities of around 90%. Furthermore, using our protocols, around 3 times more PCs and 2 times more ACs could be grown in culture, as compared to previously published protocols. The cells were identified and characterized using flow cytometry and confocal microscopy. The ability of ECs to form a tight monolayer was assessed for passages 0 to 3. The expression of claudin-5, occludin, zonula occludens-1, P-glycoprotein-1 and breast cancer resistance protein by ECs, as well as the ability of the cells to respond to cytokine stimuli (TNF-α, IFN-γ) was also investigated by q-PCR. The transcellular permeability of ECs was evaluated in the presence of pericytes or astrocytes in a Transwell^®^ model by measuring the transendothelial electrical resistance (TEER), dextran-FITC and sodium fluorescein permeability. Overall, ECs at passages 0 and 1 featured the best properties valued in a BBB model. Furthermore, pericytes did not increase tightness of EC monolayers, whereas astrocytes did regardless of their seeding location. Finally, ECs resuspended in fetal bovine serum (FBS) and dimethyl sulfoxide (DMSO) could be cryopreserved in liquid nitrogen without affecting their phenotype nor their capacity to form a tight monolayer, thus allowing these primary cells to be used for various longitudinal *in vitro* studies of the blood-brain barrier.

## Introduction

The blood-brain barrier (BBB) is composed of specialized endothelial cells (ECs) surrounded by two basement membranes, pericytes (PCs) and astrocytes (ACs) [1]. These ECs express high levels of tight junction proteins that strongly minimize paracellular diffusion and cellular transmigration in homeostatic conditions [2]. The presence of very few pinocytotic vesicles and a high concentration of efflux transporters has also been previously described on blood-brain barrier forming ECs [3, 4]. Together, those characteristics generate a physically sealed barrier allowing brain capillaries to control the passage of compounds from the blood into the central nervous system (CNS).

The BBB, due to its highly selective permeability, represents a major challenge to overcome in the development of new treatments targeting CNS diseases. In 2005, William M. Pardrige highlighted the necessity to improve our knowledge on the fundamental properties of the BBB [5] and since then, extensive studies have led to a better understanding of molecules, pathways and cells able to generate and maintain the BBB [6]. These efforts have been complemented by the design of several *in vitro* models and systems to evaluate the BBB in healthy and pathological conditions. Among these *in vitro* models, endothelial cell monocultures, co-cultures and tri-cultures with pericytes and astrocytes, either in static or dynamic culture conditions, have been described [7]. One of the caveats of these *in vitro* models resides in the fact that scientists predominantly rely on immortalized cell lines, which can deviate significantly from their *in vivo* counterparts in terms of morphology and intrinsic characteristics. Furthermore, careful interpretation of previously published results is warranted due to the use of contaminated cell lines by other cell types and in some cases, the misidentification of the original cells used to generate the cell lines [8, 9].

Alternatively, isolated primary ECs, PCs and ACs used in *in vitro* models offer several advantages compared to cell lines and *in vivo* studies. The combination of primary cells allow the study of intra- and intercellular interactions without the complexity of all cellular and molecular players normally involved *in vivo* [10]. Furthermore, contrary to immortalized cell lines, primary cells are known to keep most of their *in vivo* characteristics. As such, *in vitro* models based on primary cells isolated from the BBB offer a reliable means to evaluate drug delivery [7], immune cell infiltration [11] or protein expressions under pathological conditions [12].

Primary BBB cells have been isolated from various species. The most commonly investigated originate from murine, porcine, bovine and human tissues [13]. Undoubtedly, primary human cell cultures are the best proxy to *in vivo* human cells and as such, they represent the best way to expand our knowledge of the human BBB and improve treatment of human CNS diseases. Nonetheless, mouse primary cells are still the most used in scientific studies investigating human pathologies, mainly due to their availability [7]. Indeed, mice are the most used laboratory animals worldwide and the completion of the first draft sequence of its genome in 2002 facilitated the generation of genetically-modified strains, allowing the investigation of gene-specific functions [14, 15].

In this study, three protocols used to isolate, enrich and characterize primary mouse endothelial cells, pericytes and astrocytes are presented. ECs cells were isolated from adult mouse brains (between 6 and 15 weeks old), whereas PCs and ACs were obtained from newborn mice (3–5 days old). After mechanical dissociation and enzymatic digestion, fragments of brain microvessels were used to generate enriched cell cultures. The presence of cell-specific markers was assessed in each cell type using flow cytometry and confocal microscopy. EC characteristics were further studied by measuring their ability to produce a tight monolayer and to react to cytokine stimuli. Furthermore, primary ECs were able to survive cryopreservation and display similar low permeability post-thawing, as compared to freshly isolated primary ECs. This study aims to underline various essential characterizations to be performed on freshly isolated BBB primary cells to guarantee their suitability for further investigations.

## Materials and methods

To abridge the text, all reagents, consumables and equipment used in this study are listed in S2, S3 and S4 Tables, respectively. This study was approved by Comité de déontologie de l'expérimentation sur les animaux (CDEA) of the University of Montreal. CDEA is mandated by the Canadian Council on Animal Care (CCAC). Approved protocol: 18-083-Experimentation. Mice were sacrificed by CO2 in an euthanasia chamber, strictly following the animal facility guidelines.

### Brain extractions

Brains were extracted from 6-8-week-old (n = 20) and 1-3-day-old (n = 10), wild type male and female mice (C57Bl/6). All animals were treated in compliance with the institutional requirements set out by the Comité de déontologie de l’expérimentation sur les animaux (CDEA) of Université de Montréal (protocol # 16–083). Adult mice were euthanized with CO_2_ whereas neonatal mice were laid onto a paper put on ice prior to being beheaded with scissors. Brains were stored in cold DMEM/penicillin/streptomycin (1×).

### ECs isolation and enrichment

Endothelial cells (ECs) were isolated from adult mice. Each extracted brain was separated in three parts. The brains were cut along the longitudinal fissure to separate the two hemispheres and cut between the cerebellum and the midbrain to separate the cerebellum, pons and medulla from the rest of the brain. The meninges and choroid plexuses were removed by rolling the individual parts on P8 grade filter paper. A mechanical reduction was performed by mincing the brain pieces using surgical blades and then forcing them through sterile 18G, 20G and 22G needles, in this consecutive order. The homogenate was centrifuged (800 *g*, 8 min, 4°C) and the precipitate was digested in DMEM containing 1.05 mg × mL^-1^ type II collagenase and 58.5 U × mL^-1^ type I DNase for 75 min on a benchtop orbital shaker (100 RPM) at 37°C. The reaction was stopped by adding ice-cold DMEM and the homogenate was pelleted by centrifugation (800 *g*, 8 min, 4°C). The myelin was separated by resuspending the pellet in DMEM containing 20% BSA and centrifuging (1,000 *g*, 20 min, 4°C). To maximize brain microvessels recovery, the collected supernatant and floating myelin layer underwent another centrifugation cycle (same conditions), while the pellet was kept on ice. The combined pellets were then digested in DMEM containing 1 mg × mL^-1^ collagenase/dispase and 39 U × mL^-1^ type I DNase for 60 min on a benchtop orbital shaker (100 RPM) at 37°C. During the digestion, a 33% continuous isotonic Percoll gradient was obtained by mixing 1 mL of 10x HBSS, 9 mL of Percoll, 1 mL of 1x HBSS and 1 mL of fetal bovine serum (FBS). The isotonic Percoll mix was then centrifuged (30,000 *g*, 60 min, 4°C). The digested homogenate was then washed in ice-cold DMEM and the homogenate was pelleted by centrifugation (800 *g*, 8 min, 4°C). In order to separate microvessels from brain cellular contaminants, the pellet was resuspended in 1 ml of ice-cold DMEM and overlay on top of the cold 33% continuous isotonic Percoll gradient and then centrifuged (1,000 *g*, 10 min, 4°C, slow acceleration, slow deceleration). The microvessel layer (found close to the bottom of the ultra-centrifuge tube) was collected and washed in ice-cold DMEM by centrifugation (800 *g*, 8 min, 4°C). Brain microvessels were then seeded at a dilution corresponding to one brain per well on 6-well plates previously coated for 4 h with type IV collagen (5 μg × cm^-2^). Microvessels were cultured in high glucose DMEM supplemented with 20% FBS, penicillin/streptomycin (1×), 1 ng × mL^-1^ basic fibroblast growth factor (bFGF) [16], 100 μg × mL^-1^ heparin [17], 1.4 μM hydrocortisone [18], 8.4 μg/mL Insulin/ 7.6 μg/mL Transferrin/ 10 ng/mL Sodium Selenite supplement (ITS) and 10 μg × mL^-1^ puromycin [19]. The culture medium was replaced every other day using fresh ECs culture medium containing only 4 μg × mL^-1^ puromycin. Once the cells reached 90–95% confluence, the cultures were passaged using 0.25% Trypsin/EDTA. Confluent cells, around 7 days after seeding the microvessels, were labelled as passage 0 (P0) ECs. Thereafter, the passage number was increased by one after each trypsinization.

### PCs isolation and enrichment

Pericytes (PCs) were isolated from neonatal mouse brains. While the brains were kept in cold PBS, the meninges were removed from the brains under a dissection microscope. Then, the brains were mechanically minced in the same way as mentioned above in the ECs isolation protocol and the homogenate was similarly centrifuged. The cell pellet was then digested in DMEM containing 1.05 mg × mL^-1^ type II Collagenase and 58.5 U × mL^-1^ type I DNase for 10 min at room temperature (RT). The reaction was stopped by adding ice-cold DMEM and the cells were collected by centrifugation (800 *g*, 8 min, 4°C). The cell mixture was directly cultured in T25 flasks, at a dilution corresponding to 5 brains per flask, in low glucose DMEM (to avoid the proliferation of ECs) supplemented with 20% FBS, penicillin/streptomycin (1×), 42 μg/mL Insulin/ 38 μg/mL Transferrin/ 50 ng/mL Sodium Selenite supplement (ITS), 100 μg × mL^- 1^ heparin and Smooth Muscle Growth Supplement (1x). The medium was changed two days later. Then, every other day, 50% of the volume was discarded and replaced by an equivalent volume of fresh medium. To obtain enriched pericyte cultures, the cells were trypsinized using 0.25% Trypsin/EDTA once they reached 90–95% confluence. Following centrifugation, the cells were resuspended in fresh culture medium and were left in a flask to settle for 1 h. Subsequently, adherent cells were discarded and only the cells in suspension were kept in culture. This process was repeated after every trypsinization.

### ACs isolation and enrichment

The astrocytes (ACs) were isolated from neonatal mouse cortices (other brain parts being discarded). While the brains were kept in cold PBS, the meninges were removed from the brains under a dissection microscope. A mechanical dissociation of the cortices was performed by passing the tissue through a 22G sterile needle. The homogenate was then centrifuged (800 *g*, 8 min, 4°C) and the cell pellet was digested in DMEM containing 1.05 mg × mL^-1^ type II Collagenase and 58.5 U × mL^-1^ type I DNase for 10 min at RT. The reaction was stopped by adding ice-cold DMEM and the cells were pelleted by centrifugation (800 *g*, 8 min, 4°C). The collected cell mixture was cultured in T75 flasks previously coated with poly(L-ornithine) (8 μg × cm^-2^) at a dilution corresponding to 1 brain per flask, in low glucose DMEM supplemented with 20% FBS, penicillin/streptomycin (1×), 15 mM HEPES. Once the cells reached 85–90% confluence, the cultures were passaged by using 0.25% Trypsin/EDTA.

### Cell characterization

#### Optical microscopy

Cells were observed daily under an optical microscope, with ×10 and ×20 magnifications, to monitor cell growth. Images were recorded using a ZEISS AXIOVERT S100 microscope equipped with a Moticam 3+ camera.

#### Flow cytometry assays

Flow cytometry (FACS) experiments were performed on a BD Biosciences LSR II flow cytometer using BD FACSDiva^TM^ software (v8.0.1). FACS results were then analysed using FlowJo^TM^ software (v10.0.7). Antibodies used for FACS analysis are detailed in S5 Table. Extracellular and intracellular stainings were performed as previously described [12]. Briefly, cells were trypsinized at different passages. Isolated cells were labelled with the following antibodies against surface markers: CD11b, CD45, CD31, PDGFR-β, GFAP and GLAST-1. Cell viability was assessed with a Live/Dead marker. Intracellular staining was performed using the eBioscience fixation/permeabilization kit. The nonspecific background staining was assessed using appropriate fluorochrome-matched isotype antibodies (S5 Table).

Cells positive for CD31 and negative for CD45, CD11b, PDGFR-β, GFAP and GLAST-1 were identified as endothelial cells. Cells positive for PDGFR-β and negative for CD45, CD11b, CD31, GFAP and GLAST-1 were identified as pericytes (S1 Fig). Finally, cells positive for GFAP or GFAP and GLAST-1 and negative for CD45, CD11b, CD31 and PDGFR-β were identified as astrocytes. Cells positive for CD11b, CD45int and negative for CD31, PDGFR-β, GFAP and GLAST-1 were identified as microglia.

#### Confocal and fluorescence microscopy imaging

Cells were seeded on collagen-coated Ibidi^TM^ μ-Slides VI 0.1 for ECs, iBiTreat μ-Slide VI 0.1 for PCs and poly(L-ornithine) pre-coated μ-Slide VI 0.1 for ACs, following the manufacturer’s instructions. Briefly, the cells were diluted to reach a concentration of 5 × 10^5^ cells × mL^-1^, then 30 μL of cell suspensions were filled in each channel and incubated for 1 h at 37°C and 5% CO_2_. Finally, 60 μL of medium was added in each side reservoir. When the cells reached confluence, the medium was removed and the cells were washed three times with PBS, fixed with 70% ethanol for 5 min at room temperature and then washed again three times with PBS. The cells were subsequently permeabilized with a PBS/Tween 20 (0.05%) solution for 5 min. After permeabilization, the cells were washed and blocked with 10% species-specific sera of the secondary antibody host. The blocking solution was washed away with PBS/Tween 20 (0.05%). The cells were then incubated for 1 h at RT with primary antibodies diluted in 3% serum. Following 7 washes with PBS/Tween 20 (0.05%), the cells were incubated for 45 min at RT with secondary antibodies. Finally, the cells were covered by gelvatol reagent containing Topro-3. Each experiment included negative controls, *i*.*e*. cells incubated with secondary antibodies only. Primary and secondary antibody information are listed in S6 Table. Confocal fluorescence acquisition was performed using a Leica Confocal Microscope SP5 platform (Leica Microsystems). Fluorescence images were obtained with a fluorescence microscope Olympus IX81 (Olympus Corporation) equipped with a Retiga 2000R CCD camera (QImaging, Canada). Images were acquired with MetaMorph Advanced software (version 7.8.9.0). Image processing and analysis was performed using Image J (version 1.51).

### Cellular functionality

#### Cell impedance

ECs, PCs and ACs (P1) were seeded at 4.5 × 10^5^ cells/well on two 8W10E+ gold electrode arrays (Applied Biophysics, Troy, NY), pre-coated with collagen type IV (5 μg × cm^-2^) for ECs, and with poly(L-ornithine) (8.0 μg × cm^-2^) for ACs. Cells from each cell type were seeded in 4 wells whereas the fifth (uncultured) served as a negative control. The cells were cultured in their specific media, as previously described. The impedance of the cell monolayers was measured until a plateau was reached. The impedance measurements were then recorded at 4,000 Hz over a 72h period at a rate of 16 measurements per hour. For inflammatory conditions, primary endothelial cells at passage 0 and passage 1 were allowed to grow until the impedance reached a plateau, then the medium was replaced by fresh medium containing 100 U × mL^-1^ of IFN-γ and TNF-α. The impedance measurements were then recorded for the following 36h. The relative resistance was calculated by subtracting the resistance reached during the plateau phase by each resistance following treatment.

#### Real-time polymerase chain reaction (qPCR)

ECs (P0) were used on day 7 of culture, at a concentration of 1 brain/well in four 6-well plates, whereas ECs (P1) were used seven days after cell seeding at 1 × 10^5^ cells/well in four 6-well plates. The confluent ECs monolayers were treated with 100 U × mL^-1^ of IFN-γ and TNF-α in fresh medium or fresh medium alone as negative control. Twenty-four hours later, the cells were harvested and the mRNA isolated. qPCR was performed as previously described [20] and following Pfaffl method [21]. Briefly, total mRNA was extracted from the primary culture of ECs using RNeasy Mini Kit and transcribed into cDNA using QuantiTect Reverse Transcription kit, according to the manufacturer’s instructions. The cDNA was quantified using Applied Biosystems ViiA 7. The primer sequences used are listed in S7 Table. Relative quantification (RQ) was first calculated for each gene of interest (GOI) following Eq 1. Then, a normalized factor (NF) was calculated following Eq 2, using three housekeeping genes: hypoxanthine phosphoribosyltransferase (*Hprt*), glyceraldehyde-3-phosphate dehydrogenase (*Gapdh*) and TATA-box binding protein (*Tbp*). Finally, normalized relative quantifications were computed for each GOI following Eq 3.

$${RQ}_{GOI}=2^{{CT}_{calibrator}-{CT}_{condition}}$$ (1)

$${NF}_{experiment,sample}={\left({RQ}_{Hprt}+{RQ}_{Gapdh}+{RQ}_{Tbp}\right)}^{1/3}$$ (2)

$${NRQ}_{GOI}=\frac{{RQ}_{GOI}}{{NF}_{experiment,sample}}$$ (3)

#### Permeability

Twenty-four well Transwell^®^ Costar inserts were pre-coated with collagen type IV (5 μg × cm^-2^) or with poly-L-ornithine (8 μg × cm^-2^). Five different setups were investigated: EC monolayer, EC/PC bi-culture, with PCs seeded at the bottom of the wells (B) or on the reverse side of the insert (R) and EC/AC bi-culture, with ACs seeded at the bottom of the well (B) or on the reverse side of the insert (R). First, ACs or PCs were seeded at a density of 3 × 10^4^ cells × cm^-2^, and then the inserts were seeded with ECs at a final density of 1.5 × 10^5^ cells × cm^-2^. All cells were cultured in their respective media until ECs were seeded, then only the ECs medium was used until the cells reached confluence. When necessary, the inserts were flipped over for 24 h to allow cells to adhere. The inserts were then replaced in the plate and seeded with ECs. For experiments with cells seeded at the bottom of the wells, ECs were added on the inserts 24 h after ACs or PCs seeding. The medium was changed every 2–3 days. The permeability tests were performed on day 7 after ECs seeding. The permeability of the different *in vitro* models was assessed using fluorescein dextran (FITC-Dextran 4 kDa or 150 kDa) and sodium fluorescein (NaF). To perform the permeability assay, the culture medium was removed from the upper and bottom chambers and replaced with 100 μL of FITC-Dextran 1 mg × mL^-1^ or NaF 100 μg × mL^-1^ diluted in X-DMEM and 600 μL of X-DMEM alone, respectively. After 200 min for FITC-Dextran or 60 min for NaF, 10 μL from the top chamber medium was sampled, as well as 100 μL from the bottom chamber medium. The concentration of FITC-dextran and NaF in each compartment was calculated by measuring the fluorescence using a Saffire plate-reader (Tecan, Canada) and comparing the values to a pre-established calibration curve in X-DMEM. Excitation/emission wavelengths were set at 492/518 nm. The apparent permeability (Papp) was calculated using Eq 4 [22],

$$P_{app}=\frac{\frac{dQ}{dt}}{A\times C_{0}}$$ (4)

where Papp is the apparent permeability (cm × s^-1^), dQ/dt is the quantity of dextran (mg) in the bottom chamber at any given time, A is the insert surface area (cm^2^), C_0_ is the initial concentration of dextran in the top chamber (mg × cm^-3^).

#### TEER measurement

The transcellular resistance of the *in vitro* BBB models was measured by transferring the individual inserts in the Endhom-6 cup (World Precision Instrument, FL, United States) previously filled with 800 μL of medium. Values were recorded using the Milli ERS voltmeter (Millipore, MA, United States). The transendothelial electrical resistance (TEER) value (Ω × cm^2^) was computed by subtracting the resistance (Ω) of the filter alone to each condition and multiplying the result by the total surface of one well (0.33 cm^2^) (Eq 5). The TEER values of each cell type combinations were measured before the dextran permeability tests on the same day.

$$TEER=({Resistance}_{condition}-{Resistance}_{filter})\times {Surface}_{insert}$$ (5)

### Endothelial cell cryopreservation

Freezing procedure: freezing media were prepared by using either EC culture medium or FBS supplemented with 10% v/v sterile dimethyl sulfoxide (DMSO). Trypsinized P0 ECs were centrifuged (800 *g*, 8 min, RT) and counted using a Malassez’ counting chamber. The cells were centrifuged again and resuspended in freezing media to reach a final cell concentration of 1× or 2×10^6^ per mL. The cell suspensions were subsequently rapidly transferred into 1-mL cryovials and placed into a freezing container (Mr Frosty^™^, Thermo Fisher) at -80°C for 24 h. Finally, cryovials were transferred into liquid nitrogen for at least 48 h.

Thawing procedure: cryovials were removed from the liquid nitrogen and rapidly hand-warmed before being transferred into sterile tubes containing warm culture medium (8 mL). The tubes were then centrifuged (800 *g*, 8 min, 4°C) and the pellets were resuspended in warm culture medium.

Viability assessment: the cell suspensions were mixed with Trypan blue solution (1:1) and cells were counted twice by two independent operators (n = 4 counts) with a haemocytometer (Malassez model). Eq 6 was used to determine the cell viability with Trypan blue.

$$cell\;viability\;(\%)=100\times \frac{concentration\;of\;viable\;cells\;after\;thawing}{concentration\;of\;cryopreserved\;cells}$$ (6)

### Data statistical analyses

Statistical analyses were performed using PRISM Graphpad^TM^ software. Underlying assumptions of the ANOVA and student’s test were verified: (i) the normality of data distribution; (II) the homoscedasticity of all data subgroups. The normality of data distribution was confirmed by Q-Q plots. Where homoscedasticity was evaluated using Bartlett’s test and Brown-Forsythe’s test (α = 0.05). When tests rejected homoscedasticity multiple comparison procedures of Man-Whitney’s test (α = 0.05) were applied to identify the sample means that were significantly different from each other. Otherwise an unpaired t-test with Welch’s correction or Tukey’s test was applied. Significant results are marked with * following those rules: * P-value < 0.05, ** P- value ≤ 0.01, *** P-value ≤ 0.001. Data are expressed as mean ± standard deviation (SD).

## Results

### Isolated BBB cells express cell-specific lineage markers

**Fig 1:**
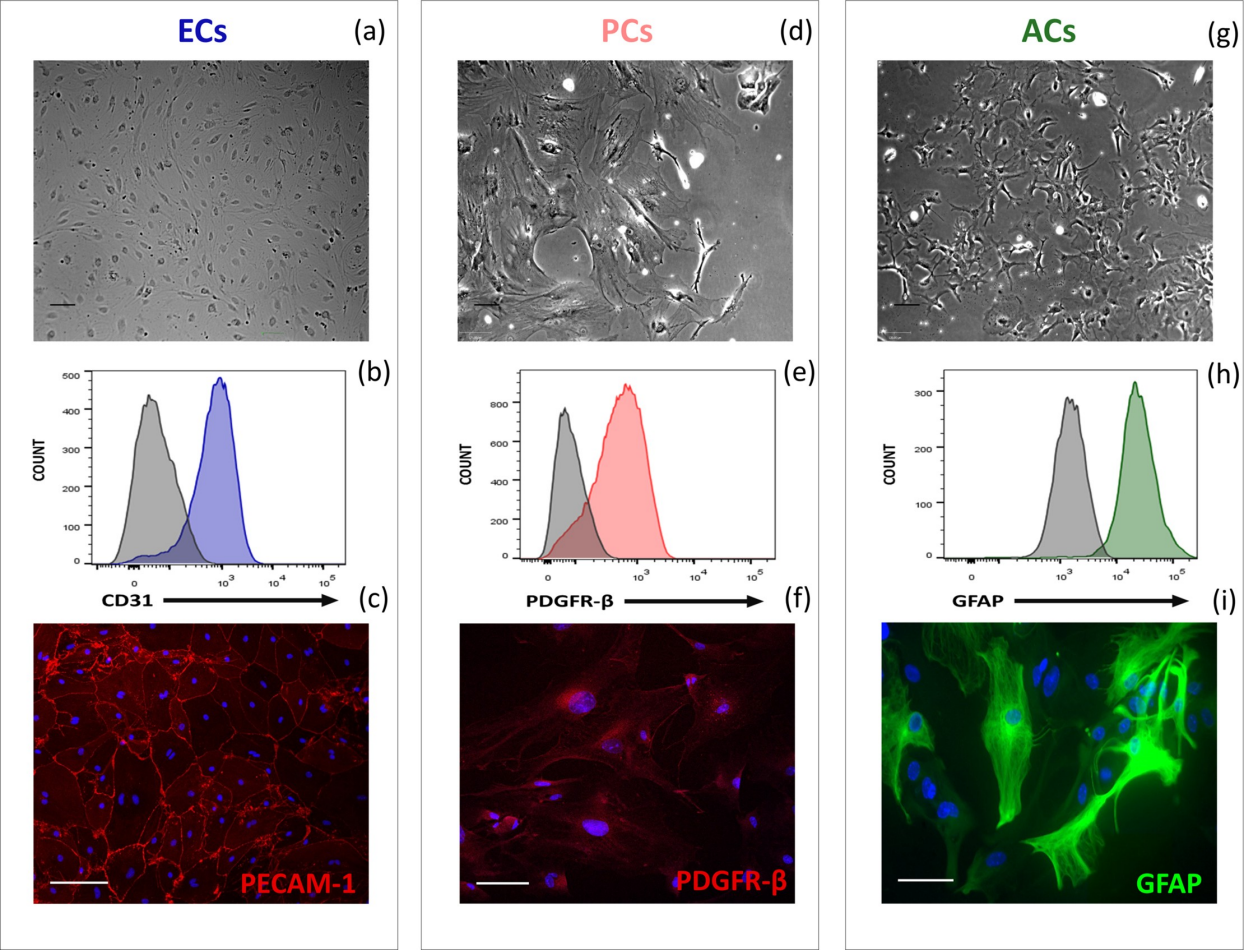
Identification of isolated brain cells from mice. (a, b, c) Endothelial cells (ECs) at P1; (d, e, f) Pericytes (PCs) at P3; (g, h, i) Astrocytes (ACs) at P1. (a, d, g) Differential interference contrast (DIC) images (× 20) of the above-mentioned cell types. (b, c) Platelet endothelial cell adhesion molecule 1 (PECAM1, CD31), (e, f) beta-type platelet-derived growth factor receptor (PDGFR-β) and (h, i) glial fibrillary acidic protein (GFAP) protein expression as assessed by (b, e, h) flow cytometry and (c, f, i) immunofluorescence confocal microscopy (× 40). Representative of n = 5. (b, e, h) Mean fluorescence intensity plots (control isotype in grey) are shown. (c, f, i) Nuclei were stained with DAPI (blue). Scale bars represent 100 μm.

The purity of the enriched primary cell cultures was assessed by flow cytometry (Fig 1B, 1E and 1H). ECs (Passage 1, P1) were identified as CD31^+^/PDGFR-b^-^/GFAP^-^/GLAST-1^-^/CD45^-^/CD11b^-^ cells, PCs (P3) were identified as PDGFR-b^+^/CD31^-^/GFAP^-^/GLAST-1^-^/CD45^-^/CD11b^-^ cells (S1 Fig) and ACs (P1) were identified as GFAP^+^/GLAST-1^+^ or GFAP^+^/GLAST-1^-^ and PDGFR-b^-^/CD31^-^/CD45^-^/CD11b^-^ cells.

ECs in culture were able to form a tight monolayer, presenting a characteristic cobblestone-like shape (Fig 1A). A majority of the cells expressed platelet endothelial cell adhesion molecule 1 (PECAM-1/CD31), as confirmed by FACS analysis, resulting in a culture purity of over 90% at P1 (Fig 1B, S2A Fig). Using 10 μg × mL^-1^ of puromycin instead of 4 μg × mL^-1^ in the enrichment medium for the first two days improved the purity of the EC cultures (S2C Fig). Contaminants were mostly identified as microglia and ACs (S2B Fig). PECAM-1 proteins were located on the EC membrane, predominantly at the intercellular junction under homeostatic conditions, as outlined by confocal microscopy (Fig 1C). Two other adhesion proteins previously characterized on ECs, namely activated leukocyte cell adhesion molecule (ALCAM) and intercellular adhesion molecule-1 (ICAM1), were also observed by confocal microscopy (S3 Fig). Junction molecules, involved in maintaining the intercellular barrier characteristic of BBB vessels, such as claudin-5 (Cldn5), vascular endothelial-cadherin (VE-cadh; CD144/cadherin-5), occludin (Ocln), zonula occludens-1 (ZO-1), zonula occludens-2 (ZO-2) and junctional adhesion molecule-A (JAM-A) were also expressed by the primary ECs in culture, as demonstrated by confocal microscopy (S3 Fig) [2].

In contrast, isolated PCs were rectangular in shape and formed an irregular monolayer (Fig 1D). The expression of platelet-derived growth factor receptor beta (PDGFR-β/CD140b) was confirmed by flow cytometry, for a culture purity of 90% at P3 (Fig 1E, S2A Fig). Contaminants were mostly identified as microglia and ACs (S2B Fig). As expected for pericytes, PDGFR-β was found uniformly distributed throughout the cell membrane (Fig 1G). Neural/glial 2 (NG2), melanoma cell adhesion molecule (MCAM) and a weak expression of alpha smooth muscle actin (aSMA) were also observed in primary PCs in culture (S4 Fig). The expression of these four biomarkers in conjunction with the absence of fibroblast-specific protein 1 (FSP1, S5 Fig) is commonly accepted to firmly identify cells as pericytes [23, 24].

Finally, ACs were found to be star-shaped when observing sub-confluent monolayers, spreading on flask bottoms with a tendency to overlap each other (Fig 1G). FACS analysis at P1 showed that a majority of the isolated cells expressed glial fibrillary acid protein (GFAP) (87%, Fig 1H, S2A Fig). Moreover, 33 to 87% (culture dependant) of GFAP^+^ cells also expressed glutamate aspartate transporter 1 (GLAST-1). GFAP distribution in the cells revealed fibrillary protein structures (Fig 1I), characteristic of this cytoskeleton component widely used as an astrocyte lineage marker [25]. The expression of GLAST-1 and S100 calcium-binding protein B (S100b), two other proteins studied in astrocytes, was also confirmed by confocal microscopy [26] (S4 Fig).

### Isolated primary ECs display blood-brain barrier characteristics

**Fig 2:**
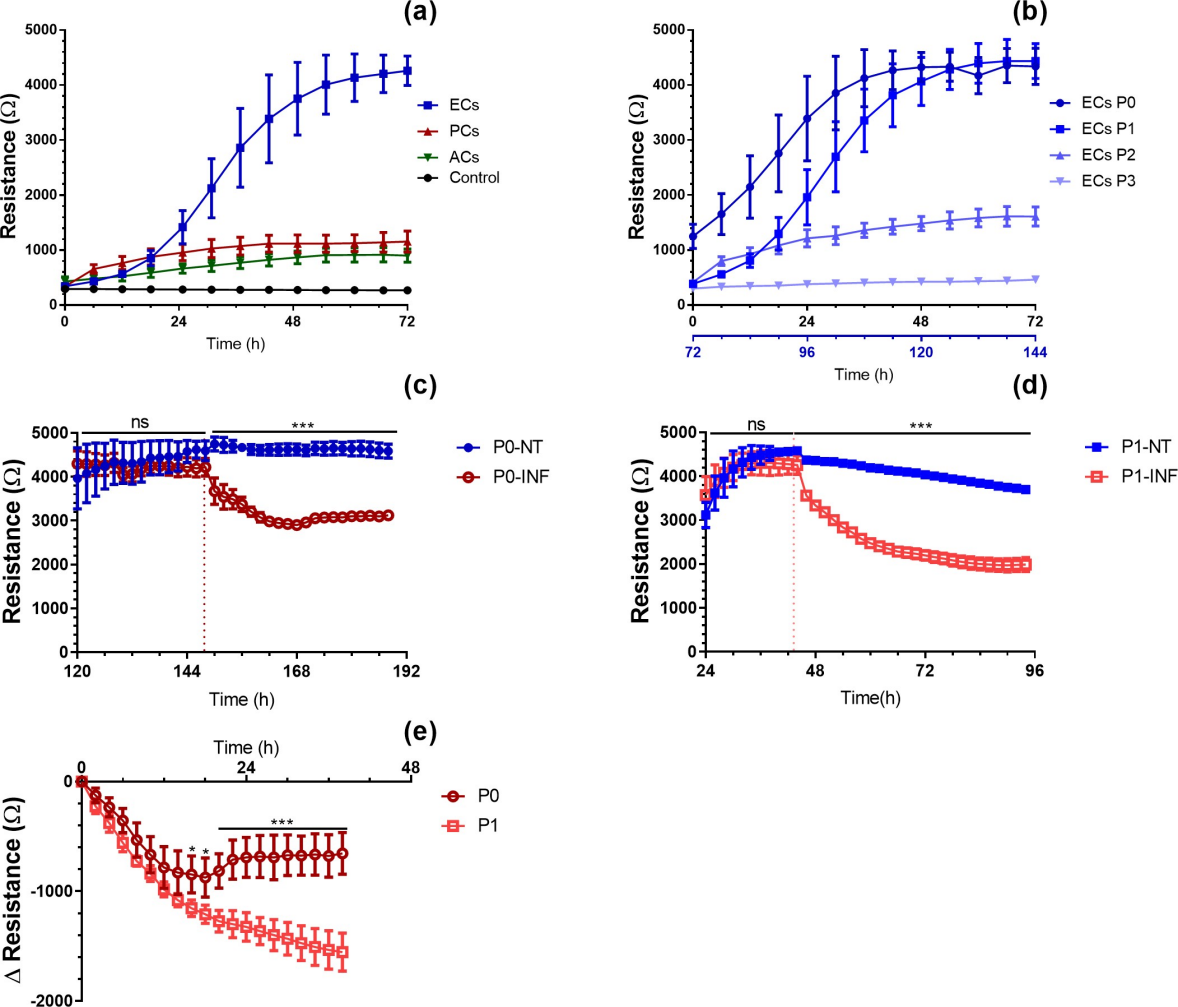
Electrical resistance of primary cultures of pericytes, astrocytes and endothelial cells, as measured by ECIS Zθ. (a) Transcellular electrical resistance comparison between pericytes (PCs), astrocytes (ACs) and endothelial cells (ECs) at passage 1 (n = 4). (b) Transendothelial electrical resistance measured from P0 to P3. The second x-axis in (b) is used to compare ECs (P0) to the other passages at similar resistance values (n = 4). (c, d) Transcellular electrical resistances of confluent monolayers of ECs at (c) passage 0 (P0) or (d) passage 1 (P1) either inflamed with 100 U/ml of TNF-α and IFN-γ (INF) or non-treated (NT) (P0: n = 5, P1: n = 4). The vertical dotted lines denote the addition of the pro-inflammatory cytokines. (e) Transcellular electrical resistance comparison of P0 and P1 ECs treated with TNF-α and IFN-γ (normalized at the treatment time point). Two-way ANOVA; * P-value ≤ 0.05, ** P-value ≤ 0.01, *** P-value ≤ 0.001. Each point represents the mean resistance measured every 6 hours (a, b) or 2 hours (c, d, e) ± standard deviations.

In addition to the qualitative analysis of specific tight junction proteins, we sought to quantify the expression of those tight junction markers and assess the permeability of isolated ECs in culture. ECs forming the CNS microvasculature are known to form an impermeable barrier, preventing most paracellular and transcellular immune cell trafficking and selectively allowing a very small number of molecules to permeate into the parenchyma under homeostatic conditions [27]. In order to assess if primary ECs in culture possess similar qualities, the impedance of the monolayers was measured, as the resistance to electron transfer from one spot of the cultured surface to another is a function of cell adherence and cell-cell junctional properties [28]. Using the Electric Cell-substrate Impedance Sensing (ECIS) technology, primary ECs were found to form a tight monolayer, displaying a high resistance of around 4,000 Ω, 120 – 144h after seeding freshly isolated brain microvessels (P0 cells) (Fig 2A and 2B & S6 Fig). To compensate for the initial growth phase of freshly isolated primary ECs as compared to trypsinized cells, P0 EC values are shown on a different axis (Fig 2B). No significant difference was observed in the maximum electrical resistance values (plateau) measured with P0 (120-144h) and P1 (48 to 72h) ECs. Importantly, ECs were not able to form a tight monolayer when passaged more than once (P2 and P3). The maximum electrical resistance values of these cell layers were found to be significantly lower than those of ECs at P0 and P1 (Fig 2B and S6 Fig). Of note, changing the culture medium appears to negatively influence the resistance of P1 cells, which could perhaps indicate a higher sensitivity to a sudden sheer-stress or to changes in CO_2_ concentration (S7 Fig). PCs and ACs, which are adherent cells involved in the formation of a functional BBB but do not form tight junctions, were used as negative controls. Both control cell types displayed significantly lower electrical resistance values, around 1100 Ω for ACs and 850 Ω for PCs (48h-72h in cultures).

### Endothelial cells cultured in inflammatory conditions

The loss of BBB integrity upon inflammation has been well studied in both *in vitro* [29, 30] and *in vivo* models [31]. It is predominantly characterized by a reduction in the expression of junctional molecules, and their apparent disorganization as well as an upregulation of cell adhesion molecules. In order to verify that the isolated primary ECs could still respond to pro-inflammatory stimuli, the cells were treated with a combination of TNF-α/IFN-γ at 100 U × mL^-1^ (Fig 2C, 2D and 2E). Both cytokines have been shown to upregulate the expression of several adhesion molecules on ECs and to disrupt tight junctions, promoting leukocyte trafficking through the BBB and playing a crucial role in several pathologies such as neuroinflammation, infections and cancers [20, 32]. ECs at P0 and P1 showed similar behaviours upon inflammation, characterized by a rapid and persistent reduction of the transcellular electrical resistance (Fig 2C and 2D). However, ECs at P0 were able to partially recover after 24h with a relative resistance around– 800 Ω (as compared to values prior to inflammation), whereas the relative resistance of ECs at P1 constantly decreased, with a minimum value measured around– 1600 Ω at the end of the recorded session (Fig 2E), denoting perhaps an increased sensitivity to pro-inflammatory cytokines.

**Fig 3:**
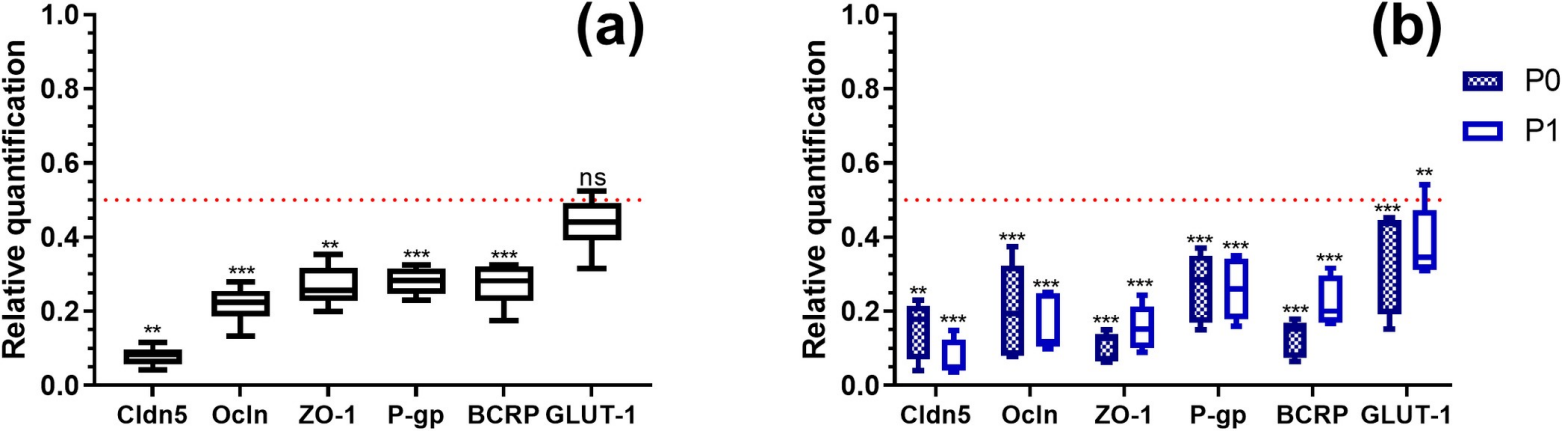
Relative quantification of the mRNA of junction and transporter proteins in primary endothelial cells. Analysis of the mRNA expression of (a) junction proteins: claudin-5 (*Cldn5*), occludin (*Ocln*), zonula occludens-1 (*Tjp1;* ZO-1) and transporter proteins: P-glycoprotein-1 (*P-gp*), breast cancer resistance protein (*Bcrp; ABCG2*) and glucose transporter-1 (*Glut-1; SLC2A1*) in endothelial cells at passage 1 (P1) assessed by q-PCR. The results are represented as relative values to P0. (b) The relative mRNA expression levels of junction and transporter proteins in P0 and P1 ECs, inflamed with 100 U/mL of TNF-α and IFN-γ for 24h. The results are relative to the corresponding untreated EC passages. All results were first normalized with *Hprt/Gapdh/Tbp* mRNA levels. The results are represented as the means ± standard deviations of 6 replicates. The dotted red lines represent a fold change of 0.5, underneath which, values were considered biologically significantly changed. Stars show significative differences between tested conditions and their corresponding base values. t-test: * P-value ≤ 0.05, ** P-value ≤ 0.01, *** P-value ≤ 0.001.

To verify the validity and relevance of such results, the mRNA expression of *Cdln5*, *Ocln* and *Tjp1* (ZO-1) from ECs at P0 and P1, under normal (untreated) and inflamed conditions was quantified using q-PCR (Fig 3). ECs at P1 showed a significant decrease in the mRNA expression levels of these junctional molecules compared to ECs at P0 (Fig 3A), albeit the differences did not strongly impact the resistance values of the cells, as previously observed (Fig 2B). A closer examination of the relative mRNA expression of the junctional molecules upon inflammation showed that both EC passages displayed similar decreased mRNA levels, as compared to untreated ECs at the same passage (Fig 3B).

Moreover, the mRNA expression of transporter proteins was also verified in similar conditions (Fig 3A and 3B). P-glycoprotein-1 (P-gp), breast cancer resistance protein (BCRP) and glucose transporter-1 (GLUT-1) were selected as representative transporters normally present on the luminal membrane of brain endothelial cells [33]. Similar to junctional molecules, a statistically significant decrease in mRNA levels of the measured transporters was observed when comparing P1 to P0 ECs as well as TNF-α/IFN-γ-treated to untreated ECs (Fig 3A and 3B).

### Bi-cellular *in vitro* BBB models impact TEER and permeability

**Fig 4:**
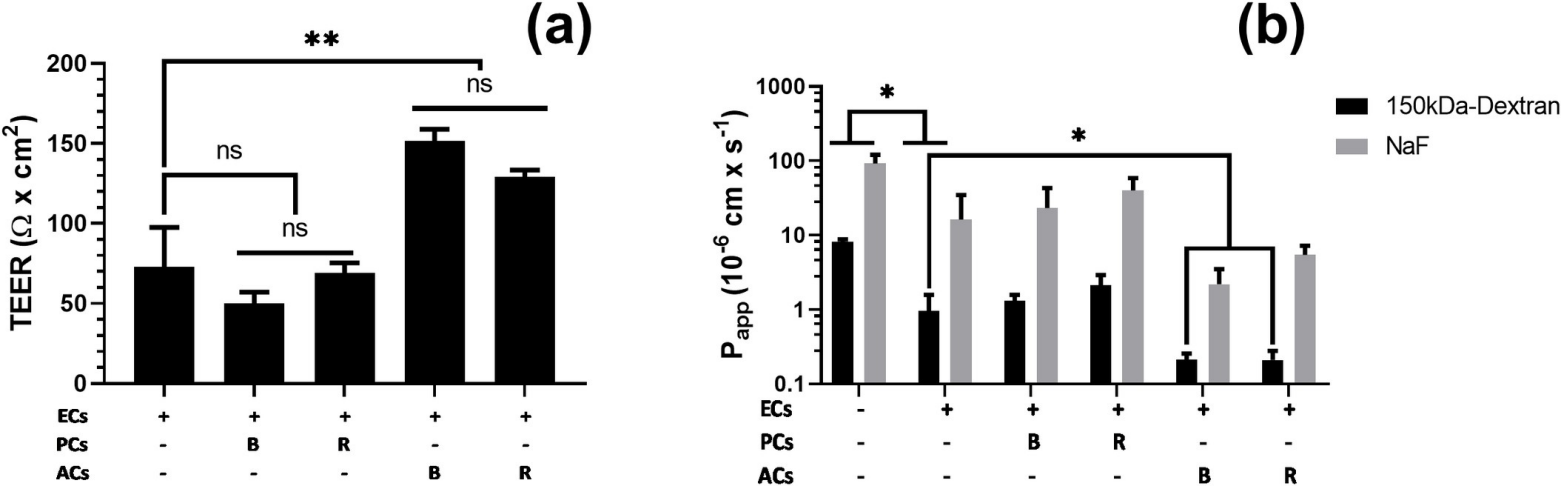
Measurement of the transcellular permeability of primary endothelial cells alone or in co-culture with pericytes or astrocytes. (a) Transendothelial electrical resistance values. (b) Permeability values of dextran-FITC (150 kDa, 1 mg/mL) and sodium-fluorescein (376 Da, 100 μg/mL). Data were acquired with primary endothelial cells (ECs) cultured on 24-Transwell^®^ in presence or absence of pericytes (PCs) or astrocytes (ACs). PCs and ACs were either cultured at the bottom of the wells (B) or on the reverse side of the inserts (mimicking the abluminal side) (R). Data represent the mean values ± SD (n ≥ 4). ANOVA with Tukey’s test: ns: non-significant, * P-value < 0.05, ** 0.001 ≤ P-value ≤ 0.01.

In order to recapitulate more closely the *in vivo* organisation of the vasculature and to assess the role of PCs and ACs in regulating the formation of a tight BBB *in vitro* model, mono- and bi-cellular cultures were studied. The transendothelial electrical resistance (TEER) and the apparent paracellular permeability (Papp) of primary EC monolayers were measured in 24-well Transwell^®^ chambers, in presence or absence of pericytes or astrocytes. Primary EC monolayers alone displayed TEER values around 70 Ω × cm^2^ and apparent permeability (Papp) values of fluorescein isothiocyanate (FITC)-dextran (Mw = 150,000 g.mol^-1^, logP ± -2) and sodium fluorescein (NaF, Mw = 376.25 g.mol^-1^, logP ± 3) around 1× 10^−6^ cm.s^-1^ and 10× 10^−6^ cm.s^-1^, respectively (Fig 4A and 4B). To assess the impact of secreted versus direct membrane-bound ligand signaling, two setups were studied: with or without contact. In contact mode (R, reverse), PCs or ACs were seeded on the reverse side of the inserts. In non-contact mode (B, bottom), PCs or ACs were seeded at the bottom of the receiver well. The EC/PC co-culture models showed no significant impact on TEER values (Fig 4A), as well as on permeability values (Fig 4B), as compared to ECs cultured alone. In contrast, both contact and non-contact models of EC/AC co-cultures were found to significantly improve TEER values (up to 130 Ω × cm^2^) (Fig 4A). In parallel, the permeability of a 150k-Dextran fluorescent marker was also decreased by a factor of five with a Papp of 0.2 × 10^−6^ cm × s^-1^ (Fig 4B). The same tendency, albeit not significantly different, was observed for the NaF marker with a Papp of 2 × 10^−6^ cm × s^-1^ (Fig 4B).

### Cryopreservation of endothelial cells

**Fig 5:**
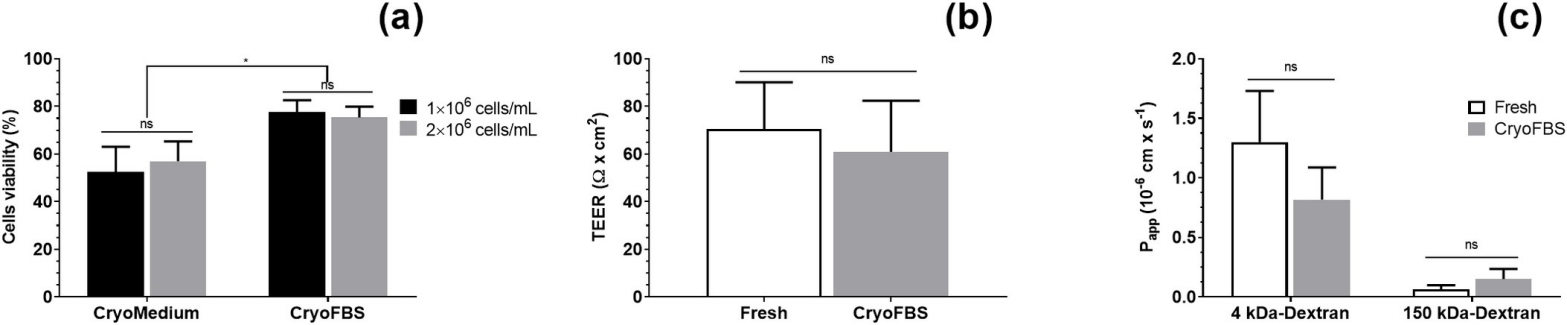
Viability of primary endothelial cells after cryopreservation. (a) Primary endothelial cells (ECs) were concentrated at 1 million cells/mL (black bars) or 2 million cells/mL (grey bars) in either ECs culture medium supplemented with 10% DMSO (CryoMedium) or foetal bovine serum supplemented with 10% DMSO (CryoFBS). Cells were counted after Trypan blue staining, prior to cryopreservation and after thawing. Results are presented as the percentage of the total number of living cells after thawing on the total number of living cells prior to cryopreservation. Data represent the mean values ± SD (n = 4). ANOVA with Tukey’s test: ns: non-significant, * P-value < 0.05. (b) Transendothelial electrical resistance (TEER) of non-cryopreserved P1 ECs (Fresh, white bar) and P1 ECs post-cryopreservation (CryoFBS, grey bar) 9 days after seeding. Data represent the mean values ± SD (n = 8). (c) Permeability values of 4 kDa and 150 kDa FITC-conjugated dextrans through non-cryopreserved P1 ECs (Fresh, white bar) and P1 ECs post-cryopreservation (CryoFBS, grey bar) 9 days after seeding. Data represent the mean values ± SD (n = 4). T-test: ns: non-significant (P-value > 0.05).

In order to widen the scope of our protocol, we investigated whether primary cells could be cryopreserved for later experiments while maintaining their above-mentioned characteristics. For this purpose, two different cryopreservation media were compared, a cryomedium consisting of EC culture medium containing 10% dimethyl sulfoxide (DMSO) (named CryoMedium) and fetal bovine serum (FBS) containing 10% DMSO (named cryoFBS). Two cell concentrations (1 and 2 × 10^6^ cells × mL^-1^) were also compared. The cell viability upon thawing was evaluated using Trypan blue staining (Fig 5A). The cells preserved in FBS supplemented with DMSO showed a significant increased viability upon thawing. Around 75% of both cell concentrations had intact membrane as compared to 60% of the cells frozen in CryoMedium. Having established the best cryopreservation medium, the expression of junctional proteins, namely Cldn5, ZO-1 and Ocln, was assessed by microscopy using P1 ECs cultured following cryopreservation at P0 in cryoFBS and compared to freshly isolated ECs at passage 1 (S8 Fig). No significant morphological differences nor relative alterations in junctional protein expression were observed between these two conditions. In addition, the TEER of cryopreserved ECs was measured at similar levels to non-cryopreserved ECs, around 60 Ω × cm^2^ (Fig 5B). Likewise, no significant differences were detected between the two groups while assessing the permeability of a 4 kDa and a 150 kDa FITC-conjugated dextran markers (Fig 5C).

## Discussion

Primary cells present numerous advantages over established cell lines. While the latter are easier to obtain, manipulate, propagate and maintain alive, they display inherent genetic deviations from their originating cells which often dramatically alter their biology [34]. Conversely, while isolating primary cells is often more expensive and time-consuming, the biology of these cells is highly similar to their *in vivo* counterparts and, as such, remain the best option for most studies.

Before selecting a primary cell isolation protocol, several aspects need to be evaluated. Most notably, yields and purities can vary widely depending on the method performed and the cost associated with some techniques can become prohibitive to specific applications. During the last two decades, a wide variety of EC, PC and AC isolation protocols have been published. While we initially tried to compare some of the most cited or recently published protocols (S1 Table), many lacked basic information, such as the total number of animals used, the yield or the purity assessed by a quantitative method. While S1 Table is not an exhaustive review of the current literature, we believe it encompasses a variety of isolation procedures which represents most of published protocols. Thus, the protocols presented herein were designed by taking into consideration the available literature, while simple steps, relatively low-cost equipment and reagent requirements were preferred in order to establish an efficient and affordable *in vitro* BBB model used for drug-screening assay. The protocols rely on enrichment steps based on known cellular properties, as opposed to a positive/negative selection strategy centered on the expression of lineage markers identified by flow cytometry or antibody coupled-magnetic beads, which incidentally generates quantitatively fewer cells [35].

Ideally, for ethical and economic reasons, it would be desirable to extract all three cell types forming the BBB from the same batch of animals. While initial tests were done in that sense, three major drawbacks were found impossible to reconcile. Astrocytes isolated from adult mouse brains displayed a very slow proliferation rate, as previously reported by others [36–40]. In contrast, postnatal ECs are known to be immature and thus, ECs isolated from adult brains are preferred to study phenomena described in fully differentiated BBB-ECs [41]. In addition, most published pericyte isolation protocols grow the cells from several subcultures originating from EC isolations [37, 42]. Yet, confluent PCs are normally obtained in 3–4 weeks, whereas ECs obtained from the same tissue are confluent in 5–7 days. As a result, ACs and, reported here for the first time, PCs were isolated from newborn mice, to ensure that enough cells would be obtained in a timely manner, whereas ECs were isolated from adult mouse brains. It should be noted that, while capillaries from spinal cords could also be used as a source of BBB cells, the limited additional number of primary cells obtained, as compared to complete brains, combined with a significant increase in time required to ensure cell purity, deterred us from using them (unpublished observations).

To increase the purity of primary EC cultures, some protocols use the selective agent puromycin, an aminonucleoside antibiotic known to be toxic to cells that do not express high levels of P-glycoprotein [19].A selective concentration of 4 to 8 μg/mL is often used, leading to reported purities of 95 to 99% [43–45]. In the protocol shown here, the initial concentration of puromycin was raised to 10 μg/mL for the first two days, followed by 4 μg/mL for the remaining time in culture. This was observed to be non-toxic for the ECs and led to a purity of 92 ± 3% at P1 (n = 5). While this value is lower than some previously reported results, it was obtained by precise and reproducible flow cytometry quantification of thousands of cells, contrary to others who deduced their purity figures from smaller sample size using fluorescence microscopy [43–45] or did not provide enrichment information altogether [46, 47]. Another important aspect of our protocol is the presence of hydrocortisone in the selective medium. This drug is known to promote tight junction formation, especially when combined with puromycin [18]. Several previously reported protocols use cell culture media that lack this corticosteroid [43, 44, 46], leading to poorer barrier properties, some with TEER values as low as 30 Ω.cm^2^ [45].

The cells obtained using our EC isolation and enrichment protocol possess a high TEER value, express key tight junction and adherens junction proteins as well as important transporter proteins, all of which are hallmarks of ECs forming the BBB. These ECs, in a similar manner to *in vivo* BBB-ECs, respond to inflammatory stimuli by upregulating adhesion molecules, downregulating junctional molecules and transporter proteins, which increases the EC barrier permeability and decreases the TEER. These characteristics, which are essential to a proper *in vitro* BBB, are seldom reported in previously published papers [48, 49]. In addition, it is well-known that primary cells deviate from their *in vivo* counterpart with each passage [50]. Herein, we clearly demonstrated that the TEER values of primary ECs at passages 0 and 1 do not significantly differ from one another, whereas we would advise against using primary ECs of higher passages. In addition, we provided conclusive evidences that cryopreserved ECs at P1 possess statistically undistinguishable barrier properties compared to freshly isolated ECs. To decrease the permeability of the *in vitro* BBB model even further and to more closely recapitulate the contribution of the intricate intercellular signalling of the neurovascular unit, we also tested different co-culture models. The addition of primary ACs to the reverse side of the Transwells or to the bottom of the wells improved significantly the barrier properties of the *in vitro* model.

In this study, ACs were isolated from newborn mice between 2- and 5-day old, with a final isolation yield of around 4 million astrocytes per brain at P1, which is higher than previously published protocols. One-day-old newborns provided lower yields, while the cell proliferation was significantly hampered using 5-day-old or older mice, as previously reported by others [39, 40]. Feldmann *et al*. and Schildge *et al*., which used slightly different isolation protocols, reported final yields of 1 and 2 million astrocytes per brain, respectively [51, 52]. Contrary to many protocols, our isolation technique did not include a filtration step through a 70/100-μm mesh [40, 51] and relied on a type II collagenase solution, leading to better cell dissociation compared to that of trypsin [40, 51, 52]. According to Josep Saura, AC cultures are repeatedly shown to be composed of approximately 90% astrocytes and 10% microglia, rendering the final cell suspension an astroglial-enriched culture [53]. While a 10% contamination could be an issue in biochemical or mechanistic studies, the microglia found with the ECs used in the co-culture models did not trigger any pro-inflammatory phenotypes and therefore, were considered non-problematic. If deemed necessary, microglia can easily be removed from AC cultures by shaking the flask for 2 hours at 37 degrees, 24 to 48 h after seeding the cells [53]. Despite the presence of microglia in our cultures, the enrichment of ACs remained close to values found in the literature, *i*.*e*. between 92 and 98% [51, 52].

PCs are usually isolated from adult mouse capillaries and often require multiple subsequent passages in different selective media to obtain an adequate number of cells [37, 38, 54]. In this protocol, the cells were directly grown in a selective medium, based on low glucose DMEM supplemented with a commercially available smooth muscle growth supplement. This selective medium was previously reported to inhibit EC proliferation while not being deleterious to PCs isolated from young mouse ears [55]. These culture conditions led to a confluent and highly enriched population of PCs at passage 3, with an isolation yield of around 0.5 million cells per brain. This corresponds to a 3-time improvement compared to recently published scientific articles [37, 42] and obtained from a similar number of mice. The isolated PCs expressed PDGFR-β, NG2, MCAM and aSMA, while being negative for FSP1. In combination to PDGFR-β, both FSP1- and CD13-specific antibodies could be added to a flow cytometry PC identification panel, especially if a positive selection approach is elected. Herein, carefully removing meninges from the brain and the sequential plating approach at each passage (*i*.*e*. allowing glial cells to adhere one hour then plating the remaining floating cells) seemed to be sufficient to limit contaminant cells. Our PC culture purity was around 90%, whereas cells isolated by FACS sorting, a technique that arguably allows for the highest purity, albeit at a much lower yield, was reported to lead to PC cultures in which 96% of the cells were positive for CD13 and PDGFR-β [56]. Other protocols which rely on similar selection approaches, did not report their final yields [38, 42, 57, 58]. This remained a recurrent challenge to compare our protocols to the available literature as among the numerous already-published protocols dealing with the isolation of ECs, PCs and ACs from murine brains, most of them do not provide sufficient characterization data to establish a relevant comparison with ours [37, 40, 42–44, 46, 47, 51, 52, 56].

In summary, the EC, the PC and the AC isolation and enrichment protocols, shown here, provide high quantitative isolation yields at a relatively low cost and generate high purity cell cultures ready to be used within 5 to 14 days. Furthermore, the ECs isolated were evaluated in preliminary functional assays, demonstrating their suitability to be used in an *in vitro* BBB model, preferentially in co-culture with primary ACs. Of particular interest, the primary ECs generated can be cryopreserved without hampering their ability to form a tight barrier and therefore, promote their use in longitudinal *in vitro* studies.

## Supporting information

S1 Fig Illustration of the flow cytometry analysis strategy related to the complete identification of PCs (at P3). (PDF)

S2 Fig Results of population purity by flow cytometry analysis for a given isolation. (PDF)

S3 Fig Confocal microscopy of primary endothelial cells marked for several proteins. (PDF)

S4 Fig Confocal microscopy of primary pericytes (PCs) and astrocytes (ACs) marked for several proteins. (PDF)

S5 Fig Primary pericytes (PCs) do not express fibroblast-specific protein 1 (FSP1). (PDF)

S6 Fig Maximum impedance of endothelial cells monolayers at different passages. (PDF)

S7 Fig Electrical resistance of primary cultures of endothelial cells without treatment, as measured by ECIS Zθ. (PDF)

S8 Fig Fluorescence microscopy images comparing cryopreserved (right panel) and non-cryopreserved (left panel) primary endothelial cells for junctional protein expression.
(PDF)

S1 Table Literature protocol comparison for ECs, PCs and ACs isolation. (PDF)

S2 Table Detailed list of used reagents. (PDF)

S3 Table Detailed list of used consumables. (PDF)

S4 Table Detailed list of used equipment. (PDF)

S5 Table FACS antibodies and isotypes. (PDF)

S6 Table Confocal and ICC antibodies. (PDF)

S7 Table q-PCR probes and primers. (PDF)
