# Supplementary material for: Isolation of endothelial cells, pericytes and astrocytes from mouse brain
Source: PLoS One. 2019 Dec 18;14(12):e0226302. doi: 10.1371/journal.pone.0226302 (PMC6919623; doi:10.1371/journal.pone.0226302)
Supplement: S1 Fig — (PDF) [file pone.0226302.s001.pdf]

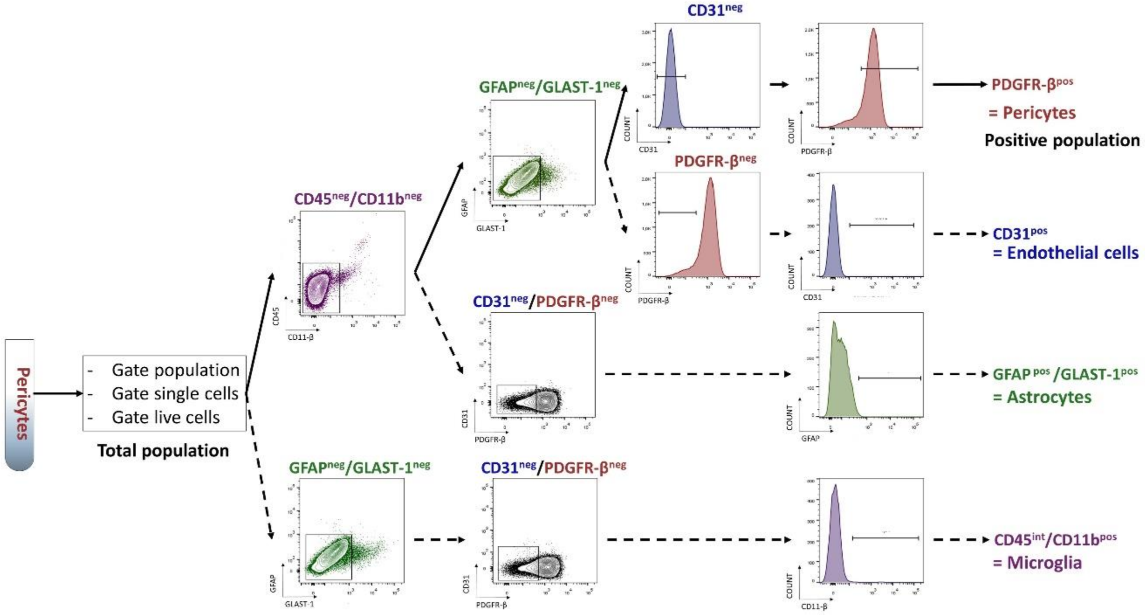

S1 Fig. Illustration of the flow cytometry analysis strategy used to identify pericytes (at P3). Protein tyrosine phosphatase receptor type c (PTPRC, CD45), integrin alpha M (ITGAM, CD11b), platelet endothelial cell adhesion molecule 1 (PECAM1, CD31) and beta-type platelet-derived growth factor receptor (PDGFR-β) were used as cell lineage markers. Similar strategies were applied for the identification of endothelial cells and astrocytes. Cells positive for glial fibrillary acidic protein (GFAP), or double positive for GFAP and glutamate aspartate transporter 1 (GLAST-1) were identified as astrocytes.

Calculation of population purity

$$Population\ purity\ (\%) = \frac{Positive\ population\ (number)}{Total\ population\ identified\ (number)} \times 100 \qquad \text{Equation (S1)}$$
