# Supplementary material for: Isolation of endothelial cells, pericytes and astrocytes from mouse brain
Source: PLoS One. 2019 Dec 18;14(12):e0226302. doi: 10.1371/journal.pone.0226302 (PMC6919623; doi:10.1371/journal.pone.0226302)
Supplement: S2 Fig — (PDF) [file pone.0226302.s002.pdf]

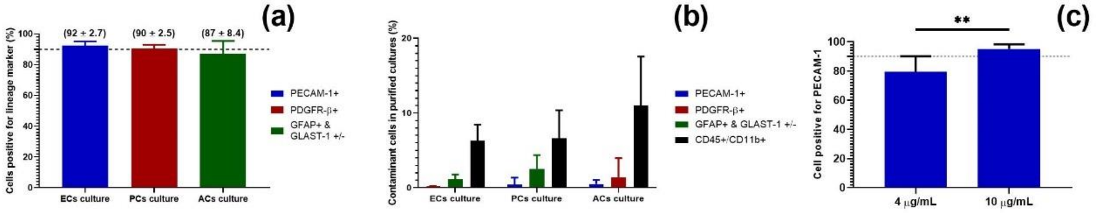


**S2 Fig. Results of population purity by flow cytometry analysis for a given isolation**. (a) Percentage of the total number of cells in culture positive for (i) blue: platelet endothelial cell adhesion molecule 1 (PECAM-1 – ECs culture at P1), (ii) red: platelet-derived growth factor receptor (PDGFR-β – PCs culture at P3) and (iii) green: glial fibrillary acidic protein (GFAP) or double positive with glutamate aspartate transporter (GLAST-1) (ACs culture at P1), respectively. **(b)** Percentage of the total number of cells in culture positive for other lineage markers (i.e.: contaminant cells) in each isolation. **(c)** Percentage of PECAM-1 positive ECs cultured with either 4 μg $\times$ mL$^{-1}$ of puromycin or 10 μg $\times$ mL$^{-1}$ in the medium for the first two days. The results are represented as the mean values ± standard deviations (n ≥ =5). T-test : ** 0.001 ≤ P-value ≤ 0.01.
