# Supplementary material for: Isolation of endothelial cells, pericytes and astrocytes from mouse brain
Source: PLoS One. 2019 Dec 18;14(12):e0226302. doi: 10.1371/journal.pone.0226302 (PMC6919623; doi:10.1371/journal.pone.0226302)
Supplement: S3 Fig — (PDF) [file pone.0226302.s003.pdf]

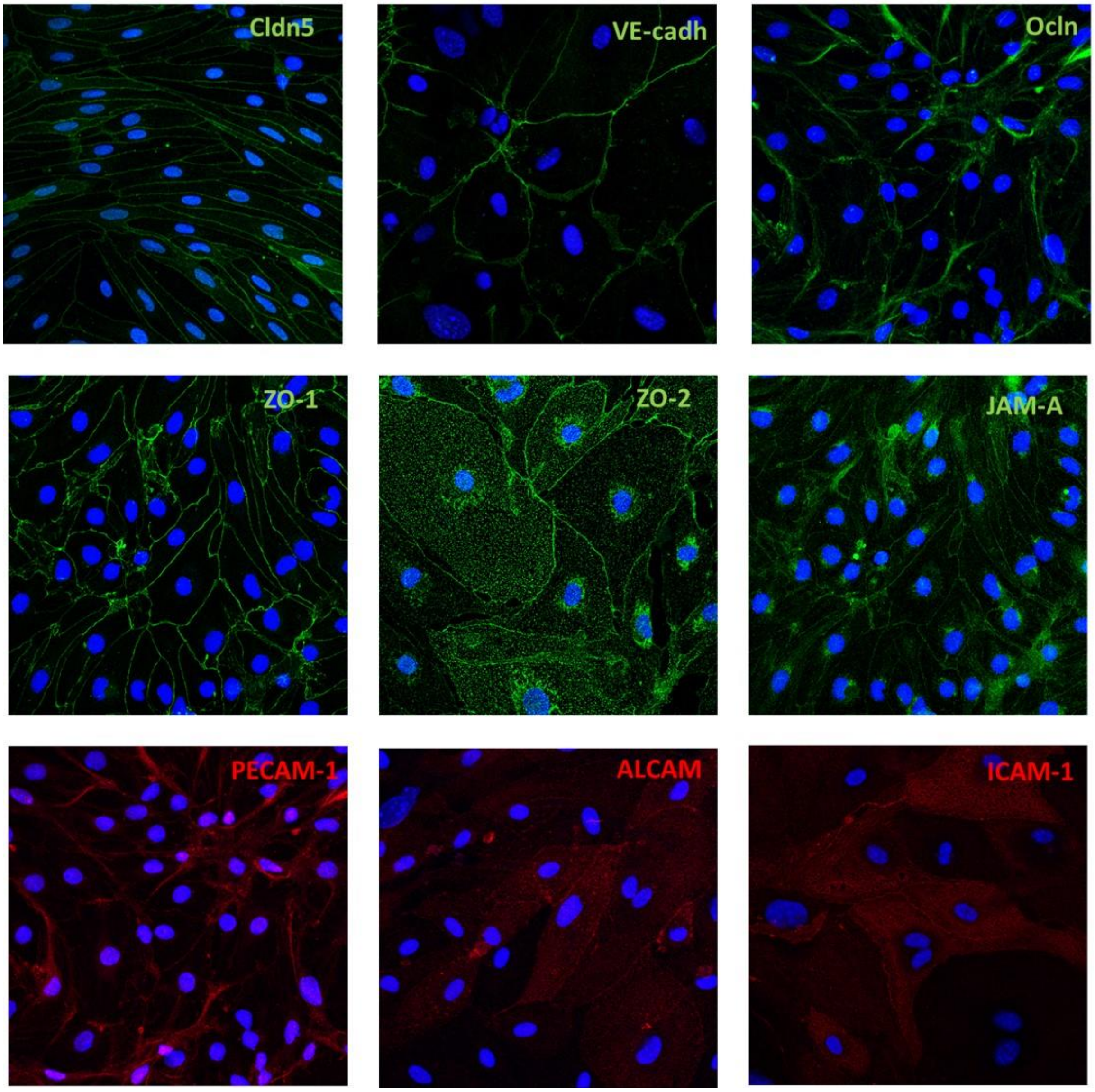

S3 Fig. Confocal microscopy of primary endothelial cells marked for several proteins. Primary endothelial cells were immunolabelled with antibodies against the following junction proteins: Claudin-5 (Cldn5), vascular-endothelial cadherin (VE-cadh), occludin (Ocln), zonula occludens-1 (ZO-1), zonula occludens-2 (ZO-2) and junctional adhesion molecule-A (JAM-A), as represented in green. Immunolabelled cell adhesion proteins, namely platelet endothelial cell adhesion molecule 1 (PECAM-1), activated leukocyte cell adhesion molecule (ALCAM) and intercellular adhesion molecule-1 (ICAM1), are shown in red. Representative of n = 4.
