# Supplementary material for: Isolation of endothelial cells, pericytes and astrocytes from mouse brain
Source: PLoS One. 2019 Dec 18;14(12):e0226302. doi: 10.1371/journal.pone.0226302 (PMC6919623; doi:10.1371/journal.pone.0226302)
Supplement: S4 Fig — (PDF) [file pone.0226302.s004.pdf]

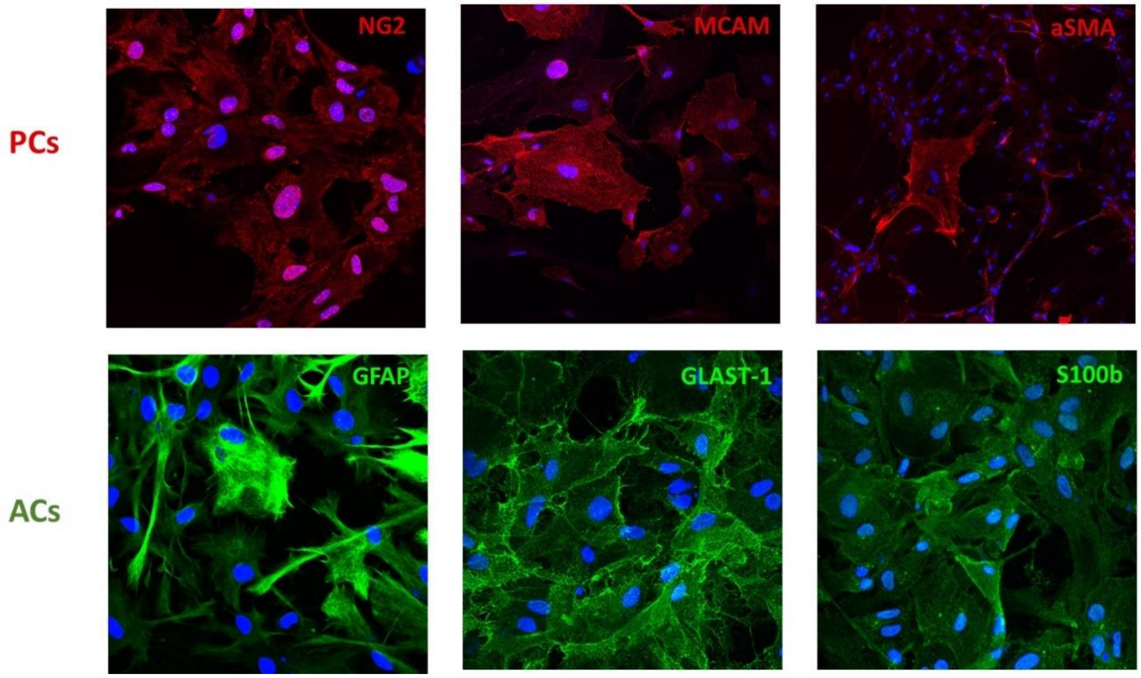

S4 Fig. Confocal microscopy of primary pericytes (PCs) and astrocytes (ACs) marked for several proteins. PCs (top panel) were immunolabelled with antibodies against neural/glial 2 (NG2), melanoma cell adhesion molecule (MCAM) and alpha smooth muscle actin (aSMA), as shown in red**.** ACs (bottom panel) were immunolabelled with antibodies against glial fibrillary acidic protein (GFAP), glutamate aspartate transporter-1 (GLAST-1) and S100 calcium-binding protein B (S100b), as shown in green. Representative of n = 4.
