# Supplementary material for: Isolation of endothelial cells, pericytes and astrocytes from mouse brain
Source: PLoS One. 2019 Dec 18;14(12):e0226302. doi: 10.1371/journal.pone.0226302 (PMC6919623; doi:10.1371/journal.pone.0226302)
Supplement: S5 Fig — (PDF) [file pone.0226302.s005.pdf]

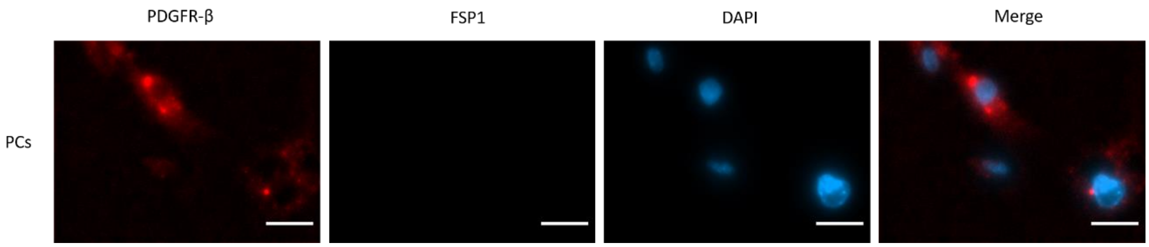

S5 Fig. Primary pericytes (PCs) do not express fibroblast-specific protein 1 (FSP1). Fluorescence microscopy images of PCs immunolabelled with antibodies against platelet-derived growth factor receptor (PDGFR-β, red) and fibroblast-specific protein 1 (FSP1, green). Representative of n = 4.
