# Supplementary material for: Isolation of endothelial cells, pericytes and astrocytes from mouse brain
Source: PLoS One. 2019 Dec 18;14(12):e0226302. doi: 10.1371/journal.pone.0226302 (PMC6919623; doi:10.1371/journal.pone.0226302)
Supplement: S6 Fig — (PDF) [file pone.0226302.s006.pdf]

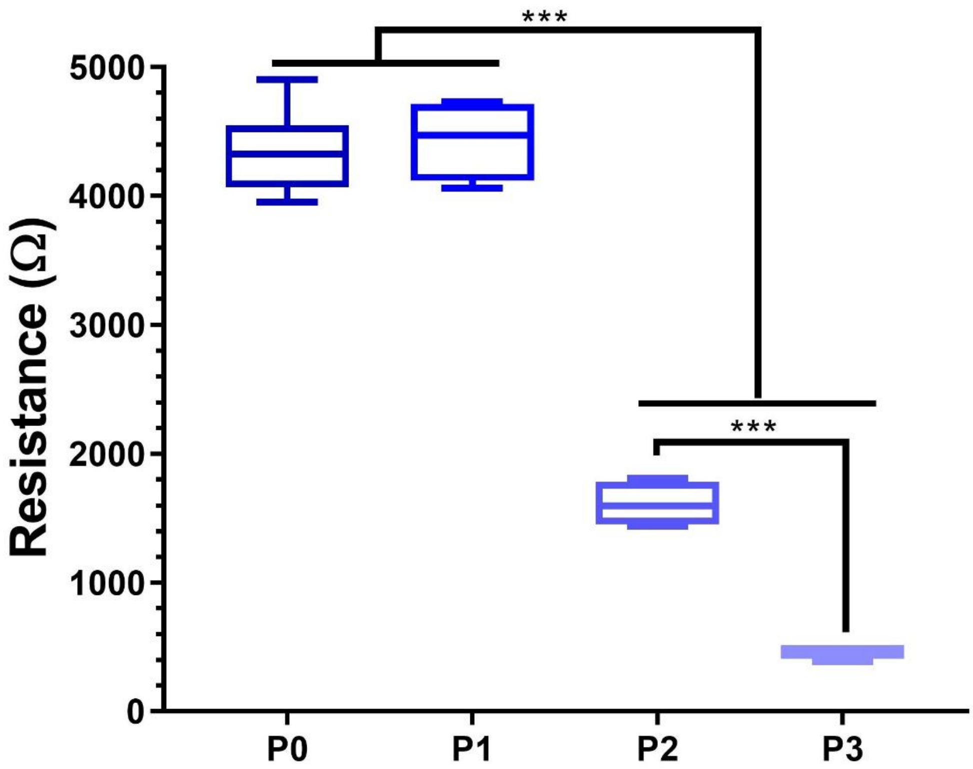

S6 Fig. Maximum impedance of endothelial cell (EC) monolayers at different passages. The measurements were done 144h after seeding primary ECs at P0 and 72h for ECs at P1-P3. The results are expressed as whiskers plots of 4 separate experiments with max, median and min values (n ≥ 4). Nonparametric Man-Whitney test: * P-value < 0.05 *** P-value ≤ 0.001
