# Supplementary material for: Isolation of endothelial cells, pericytes and astrocytes from mouse brain
Source: PLoS One. 2019 Dec 18;14(12):e0226302. doi: 10.1371/journal.pone.0226302 (PMC6919623; doi:10.1371/journal.pone.0226302)
Supplement: S7 Fig — (PDF) [file pone.0226302.s007.pdf]

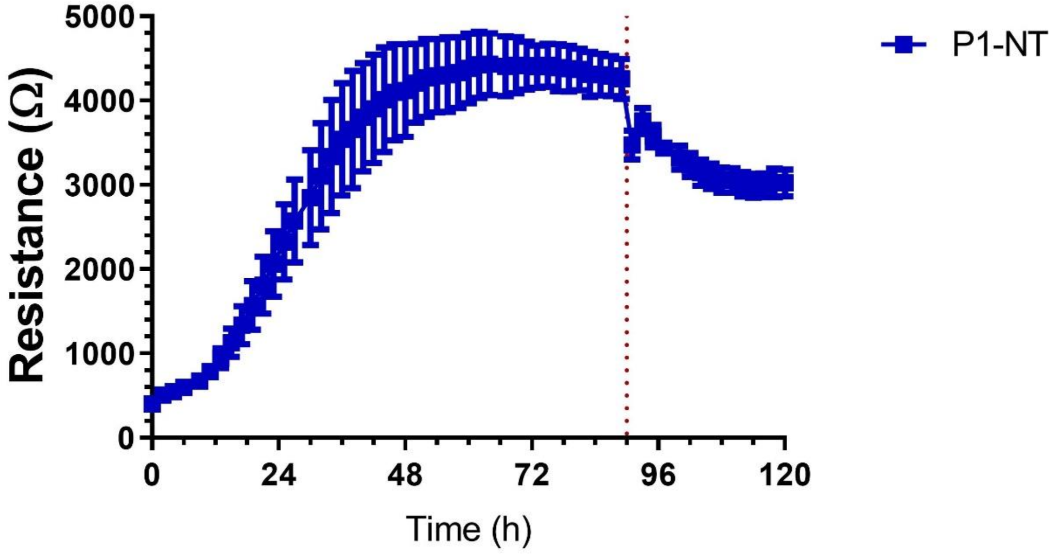

S7 Fig. Electrical resistance of primary cultures of endothelial cells without treatment, as measured by ECIS Zθ. Transcellular electrical resistance of endothelial cells (ECs) at passage 1 (n=6). The culture media was changed after 90 hours and it is represented by the dotted line. Each point represents the mean resistance measured every 2 hours ± standard deviations.
