# Supplementary material for: Isolation of endothelial cells, pericytes and astrocytes from mouse brain
Source: PLoS One. 2019 Dec 18;14(12):e0226302. doi: 10.1371/journal.pone.0226302 (PMC6919623; doi:10.1371/journal.pone.0226302)
Supplement: S8 Fig — Fluorescence microscopy images comparing cryopreserved (right panel) and non-cryopreserved (left panel) primary endothelial cells for junctional protein expression. (PDF) [file pone.0226302.s008.pdf]

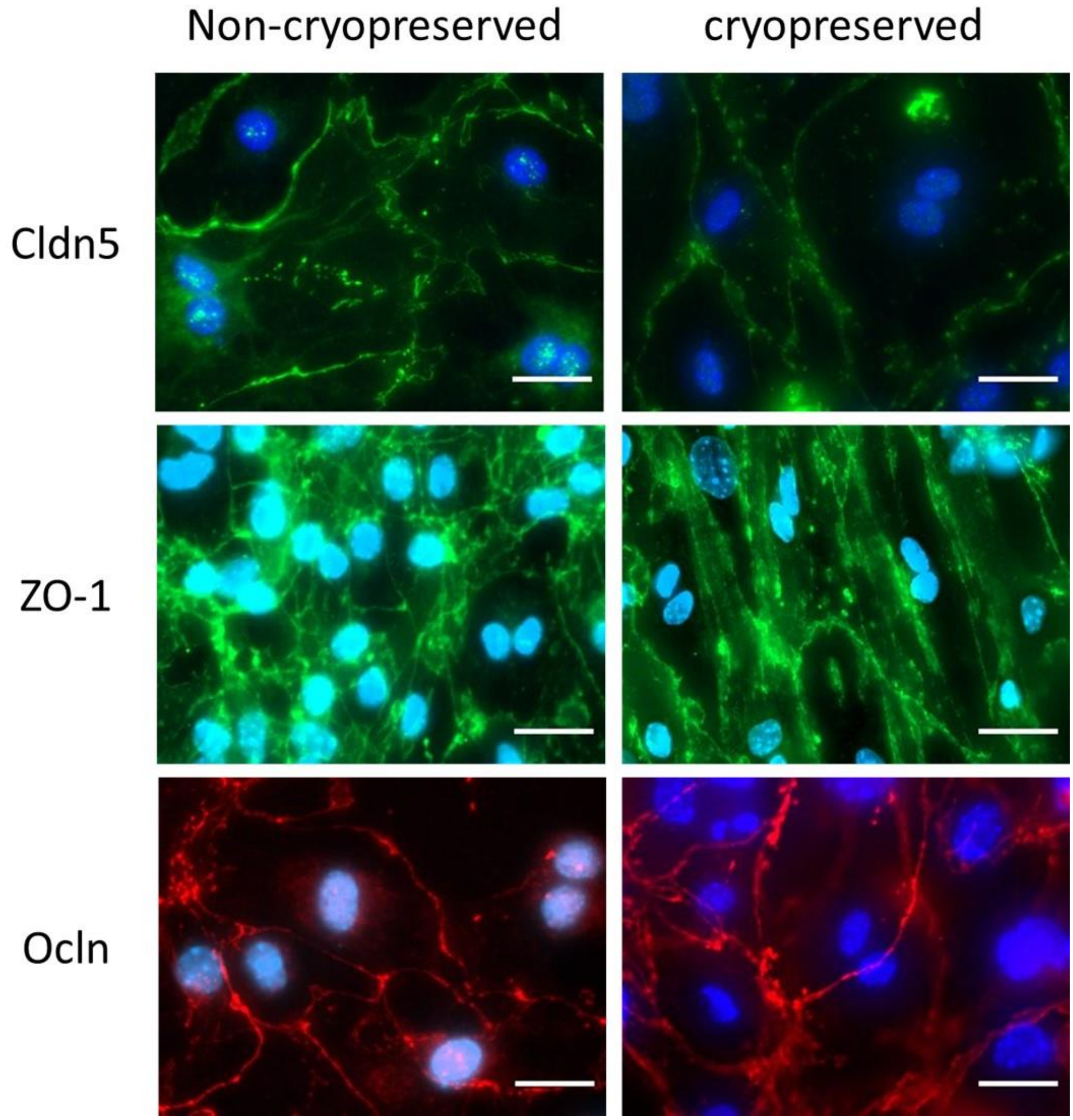

S8 Fig. Fluorescence microscopy images comparing cryopreserved (right panel) and non-cryopreserved (left panel) primary endothelial cells for junctional protein expression. Previously cryopreserved ECs (in 90% FBS and 10% DMSO at a concentration of 1 million cells/mL) currently at passage 1 (P1) (right panel) and non-cryopreserved P1 ECs (left panel) are shown. The cells in both conditions were allowed to reach confluence before imaging. Claudin-5 (Cldn5) (top), zonula occludens-1 (ZO-1) (center) and occludin (Ocln) (bottom) were immunolabelled and imaged using a fluorescence microscope. Nuclei were stained with DAPI. Scale bar is 25 µm. Representative of n = 3.
