# Supplementary material for: Isolation of endothelial cells, pericytes and astrocytes from mouse brain
Source: PLoS One. 2019 Dec 18;14(12):e0226302. doi: 10.1371/journal.pone.0226302 (PMC6919623; doi:10.1371/journal.pone.0226302)
Supplement: S1 Table — (PDF) [file pone.0226302.s009.pdf]

S1 Table. Literature protocol comparison for ECs, PCs and ACs isolation

Cell type	Ref DOI	Year	Methods	Species	Number of mice	Mice Age	Mechanical reduction	Meninges removal	Enzymatic reduction	Myelin removal method	Selecting media	Microvessels extraction time	cell selection duration	Collagen concentration	Number of passage before use	Isolation yield (cells/brain)	Purity (%) (method)	Tight junction proteins	Adherens junction proteins	Other proteins	TEER (passage, instrument, values)	Permeability	Models assembly	cryopreserved / functionality assessment
ECs	Bernard-Patrzynski F. *et al*	2019	Enrichment	C57BL/6	20	6-12 weeks	Razor blade, syringe	Yes	1.5 X collagenase II (1h15) + collagenase/dispase/ DNAse I (1h)	20 % BSA x2	10 and 4 ug/mL Puromycin, heparin, bFGF, IPS, hydrocortisone + collagen IV	5 h	6-8 days	5 µg/cm²	1	1.5×10^5	92 % (FACS)	Ocln, ZO-1, Cldn-5, ZO-2, JAM-A	VE-cadherin	PECAM-1, ALCAM, ICAM, P-gp, BCRP. GLUT-1	Endhon cup, P0 to P3, 4000 ohm	4kDa and 150kDa-Dextran	ECS-PCs / ECS-ACs	Viability / microscopy
ECs	10.21769/BioProtoc.3091	2018	Enrichment	N.A.	2	N.A.	Razor blade, filtering 70 µm	Yes	Enzyme from company NDK (25 min)	0.9 M sucrose	N.A.	N.A	N.A.	N.A.	N.A.	N.A.	N.A.	CD45	VCAM-1	PECAM-1	N.A.	N.A.	N.A.	N.A.
ECs	10.1038/nprot.2017.158	2018	Sorting	CD31 mice	N.A.	8 weeks	Razor blade, microsurgery knives	Yes	collagenase/dispase (30 min)	22 % Percoll x 1	EGM-2 media + collagen	5 h	N.A.	18 µg/cm²	N.A.	N.A.	97 % (FACS)	N.A.	N.A.	PECAM-1	N.A.	N.A.	N.A.	N.A.
ECs	10.21769/BioProtoc.2294	2017	Enrichment	C57BL/6	N.A.	6-52 weeks	Tissue grinder	Yes	collagenase/dispase/ DNAse I (1h15)	18 % Dextran x 1	Puromycin 8 ug/mL + heparin + ECGS + PDF (plasma derived fetal serum) + collagen IV	N.A	6-8 days	15 µg/cm²	1	N.A.	95 % (ICC)	Ocln, ZO-1, Cldn-5	VE-cadherin	PECAM-1	N.A.	N.A.	N.A.	N.A.
ECs	10.1002/cyto.a.22677	2015	Beads	C57BL/6 and C57BL/6 GFP+	N.A.	1 - 2 day / 10 weeks	70 µm filter syringe + 40 µm	Yes but don't explain how	Neural digestion kit + MACS dissociation or papain or nylon	MACS buffer and Myelin Removal Beads II or 30 % Percoll x1	EGM-2-MV medium + collagen type I	N.A	N.A.	N.A.	N.A.	N.A.	N.A.	CD45	N.A.	PECAM-1, CD133, CD117	N.A.	N.A.	N.A.	N.A.
ECs	10.1007/978-1-4939-0320-7_28	2014	Enrichment	N.A.	6	6-8 weeks	Razor blade, syringe	No	Papain (1h10)	22 % BSA x 1	Puromycin 4 ug /mL + heparin, 10 % FBS, L-glutamine + collagen calf skin type I	N.A	5-7 days	1.3 mg/cm²	1	N.A.	99% (ICC)	Ocln, ZO-1, Cldn-5	VE-cadherin	PECAM-1	N.A.	N.A.	N.A.	N.A.
ECs	10.3791/52204	2014	Enrichment	C57BL/6	10	8 - 12 weeks	25 and 10 mL pipette	Yes	collagenase CLS2/DNAse (1 h) + collagenase/dispase/ DNAse (1 h)	20% BSA x 1	PDS + bFGF + heparin + Puromycin + collagen IV and fibronectin + Percoll 30 %	N.A	5 days	251 µg/cm²	1	N.A.	99 % (ICC)	N.A.	N.A.	PECAM-1	20 - 30 ohm/cm2	N.A.	N.A.	N.A.
PCs	Bernard-Patrzynski F. *et al*	2019	Enrichment	C57BL/6	5	3-5 days	Razor blade, syringe	Yes	1.5 X collagenase II (1h15) + collagenase/dispase/ DNAse I (1h)	N.A.	PGM + non-coated + 20 % FBS	3h	14 days	N.A.	3	0.5×10^6	90 % (FACS)	N.A.	N.A.	PDGFR-b; SMA;NG2, FSP1 neg	N.A.	150kDa-Dextran	ECS-PCs	N.A.
PCs	10.1038/nprot.2017.158	2018	Sorting	CD1 Mice	N.A.	8 weeks	Razor blade, microsurgery knives	Yes	collagenase/dispase (30 min)	22 % Percoll x 1	EGM-2 media + collagen	5h	N.A.	18 µg/cm²	N.A.	N.A.	96 % (FACS)	N.A.	N.A.	CD13	N.A.	N.A.	N.A.	N.A.
PCs	10.1073/pnas.1710848114.	2017	Enrichment	C57BL/6J	N.A.	6 - 8 weeks	razor blade, pipette	No	Collagenase IV _ DNAse (1h30)	22 % BSA	ECGM then pericyte medium + collagen type I	N.A	N.A.	N.A.	2	3 well of 6 well plate	N.A.	N.A.	N.A.	PDGFR-b; CD146	N.A.	N.A.	N.A.	N.A.
PCs	10.1007/978-1-4939-0320-7_31	2014	Enrichment	N.A.	6	6 - 8 weeks	razor blade, pipette, syringe	No	Papain+DNAse (1h10)	22 % BSA	Endothelial cell growth supplement + Heparin + ECGM + Pericyte growth medium + Collagen	N.A	N.A.	N.A.	5	0.133×10^6	N.A.	N.A.	N.A.	PDGFR-b; CD136;NG2	N.A.	N.A.	N.A.	N.A.
PCs	10.1038/ncomms4413	2014	Enrichment	C57BL/6J	N.A.	8 – 16 weeks	homogenate	No	1 mg/mL collagenase/dispase (4h)	17 % dextran	100 and 40 µm filtered + Pericyte medium (ScienCell)	N.A	N.A	laminin-1	N.A.	N.A.	High purity	N.A.	N.A.	SMA, SM22, myocardin, PDGFR-b,	N.A.	N.A.	N.A.	N.A.
ACs	Bernard-Patrzynski F. *et al*	2019	Enrichment	C57BL/6J	5	3-5 days	Razor blade, syringe	Yes manually	collagenase II (10 min)	N.A.	Cortexes + low glucose DMEM +20 % FBS + 15 mM HEPES	3h	7 days	Poly-L-ornithine	1	4×10^6	89%	N.A.	N.A.	GFAP, GLAST-1, S100b	N.A.	150kDa-Dextran	ECs-ACs	N.A.
ACs	10.1007/978-1-4939-9068-9_3	2019	Beads	N.A.	Up to 20	1 – 3 days	Scalpel, forceps, homogenizer , 25G needle	Yes manually	N.A.	N.A.	Cortexes + 100 µm filtered + DMEM/F12 + 10 % FBS + CD11b neg selection	N.A.	10 – 14 days + beads selection	Ploy-L-lysine	1-2	5×10^5	99 % (ICC)	N.A.	N.A.	GFAP, Iba1 neg	N.A.	N.A.	N.A.	N.A.
ACs	10.1080/01616412.2018.1517995	2018	Enrichment	C57BL/6J	10	1 – 2 days		No	Trypsin (30 min)	N.A.	Cortexes + 100 µm filtered + DMEM + 10 % FBS	N.A.	14 days + shook	No	1	N.A.	N.A.	N.A.	N.A.	GFAP, Iba1 neg	N.A.	N.A.	N.A.	N.A.
ACs	10.1016/j.brainresbull.2017.05.002	2017	Enrichment	C57BL/6J	N.A.	0-4 days / 6-10 weeks	pipette	Yes manually	0.25 % trypsin (15 min)	N.A.	cortexes + 100 µm filtered + 20 % FBS DMEM + 100 µg/mL streptomycin + Forskolin 10 µM + 10 ng/mL GDNF	N.A	10 -14 days + shakers 18-20h	Gelatin + Matrigel	1	N.A.	N.A.	N.A.	N.A.	Aldoc, GFAP, S100b. Cx43, GLAST, GLT-1, CD44	N.A.	N.A.	N.A.	N.A.
ACs	10.14440/jbm.2014.33.	2014	Enrichment	CD-1 mousse	1	9-10 days	GentleMACS dissociator	No	Neural Tissue Dissociation Kit Postnatal Neurons	0.5 % BSA	70 um filtering	N.A	N.A.	N.A.	1	1×10^6	92% (ICC)	N.A.	N.A.	GFAP, ASCA-2	N.A.	N.A.	N.A.	N.A.
ACs	10.3791/50079	2013	Enrichment	N.A.	4	1-4 days	blade, pipette	Yes manually	0.25 % trypsin (30 min)	N.A.	cortexes + DMEM high glucose + 10 % FBS + poly-D-Lysine +	N.A	19 - 22 days + shaking 30 min + shaking 6 h	Poly-D-lysine	2	2×10^6	98 % (ICC)	N.A.	N.A.	GFAP, GLAST, S100B, aquaporine-4, ALDH1, BLBP	N.A.	N.A.	N.A.	N.A.
ACs	10.1016/B978-008045046-9.00867-6	2009	Enrichment	N.A.	1	1-2 days	Mechanically dissociated	Yes manually	0.25 % trypsin (20 min)	N.A.	Cortexes + EMEM + 10 % equine serum + 21 mmol/L glucose + 10 ng/mL epidermal growth factor + poly-D-Lysine +	N.A.	7-14 days	poly-D-Lysine +	1	N.A.	N.A.	N.A.	N.A.	N.A.	N.A.	N.A.	N.A.	N.A.
