# Supplementary material for: Isolation of endothelial cells, pericytes and astrocytes from mouse brain
Source: PLoS One. 2019 Dec 18;14(12):e0226302. doi: 10.1371/journal.pone.0226302 (PMC6919623; doi:10.1371/journal.pone.0226302)
Supplement: S2 Table — (PDF) [file pone.0226302.s010.pdf]

S2 Table. Detailed list of used reagents

Reagents			
**Item**	**Acronym**	**Provider**	**Catalogue #**
Bovine Serum Albumin Fraction V	BSA	Wisent, Saint-Jean-Baptiste, QC, Canada	800-095-Eg
Collagen from human placenta, type IV	Collagen type IV	Sigma-Aldrich, Oakville, ON, Canada	C5533
Collagenase, Type 2	Type 2 collagenase	Worthington Biochemical Corp., Lakewood, NJ, United States	LS004174
Collagenase/dispase	Collagenase/dispase	Sigma-Aldrich, Oakville, ON, Canada	10269638001
Deoxyribonuclease I	DNase I	Worthington Biochemical Corp., Lakewood, NJ, United States	LS002138
Dulbecco's Modified Eagle Medium high glucose	DMEM high	Wisent, Saint-Jean-Baptiste, QC, Canada	319-005-CL
Dulbecco's Modified Eagle Medium low glucose	DMEM low	Wisent, Saint-Jean-Baptiste, QC, Canada	219-020-XK
Dulbecco's Phosphate Buffered Solution, without calcium and magnesium	PBS	Wisent, Saint-Jean-Baptiste, QC, Canada	311-425-CL
Extreme DMEM	X-DMEM	Wisent, Saint-Jean-Baptiste, QC, Canada	319-100-CL
Fibroblast growth factor-Basic	bFGF	Thermo Fisher Scientific, Burlington, ON, Canada	PHG0024
Fluorescein Sodium	Na-Fluo	Sigma-Aldrich, Oakville, ON, Canada	FX0325
Fluorescein isothiocyanate carboxymethyl dextran	Dextran-Fluo	Sigma-Aldrich, Oakville, ON, Canada	74817
Fœtal Bovine Serum	FBS	Wisent, Saint-Jean-Baptiste, QC, Canada	080-150
Hank's Balanced Salt Solution (10X)	HBSS 10X	Thermo Fisher Scientific, Burlington, ON, Canada	14065-056
Hank's Balanced Salt Solution (1X)	HBSS	Thermo Fisher Scientific, Burlington, ON, Canada	14025-092
heparin sodium	heparin	Fisher Scientific, Ottawa, ON, Canada	BP2425
HEPES (1M)	HEPES	Thermo Fisher Scientific, Burlington, ON, Canada	15630-080
Hydrocortisone	Hydrocortisone	Sigma-Aldrich, Oakville, ON, Canada	H0888
Insulin-Transferin-Sodium Selenite media supplement	ITS	Sigma-Aldrich, Oakville, ON, Canada	I1884-1VL
Invitrogen, SlowFade Diamond antifade mountant with DAPI	mounting media	Thermo Fisher Scientific, Burlington, ON, Canada	S36968

Iodonitrotetrazolium chloride	INT	Sigma-Aldrich, Oakville, ON, Canada	I8377
Lithium-Lactate	Lithium-Lactate	Sigma-Aldrich, Oakville, ON, Canada	440469
Penicillin Streptomycin	Pen/Strep	Wisent, Saint-Jean-Baptiste, QC, Canada	450-201-EL
Percoll	Percoll	GE HealthCare Bio-Sciences, Baie-d'Urfé, QC, Canada	17-0891-01
Phenazine methosulfate	PMS	Sigma-Aldrich, Oakville, ON, Canada	P9625
Poly-L-ornithine	PLO	Sigma-Aldrich, Oakville, ON, Canada	P3655
Puromycin dihydrochloride from Streptomyces alboniger	puromycin	Sigma-Aldrich, Oakville, ON, Canada	P8833
Smooth Muscle Growth Supplement	SMGS	Thermo Fisher Scientific, Burlington, ON, Canada	S00725
Thiazolyl Blue Tetrazolium Bromide	MTT	Sigma-Aldrich, Oakville, ON, Canada	M5655
Tris(hydroxymethyl)aminomethane	Tris	Sigma-Aldrich, Oakville, ON, Canada	252859
Trypsin-EDTA (0.25 %), phenol red	Trypsin	Thermo Fisher Scientific, Burlington, ON, Canada	25200072
β-Nicotinamide adenine dinucleotide sodium salt	NAD	Sigma-Aldrich, Oakville, ON, Canada	N0632
