# Supplementary material for: Isolation of endothelial cells, pericytes and astrocytes from mouse brain
Source: PLoS One. 2019 Dec 18;14(12):e0226302. doi: 10.1371/journal.pone.0226302 (PMC6919623; doi:10.1371/journal.pone.0226302)
Supplement: S3 Table — (PDF) [file pone.0226302.s011.pdf]

S3 Table. Detailed list of used consumables

Consumables			
**Item**	**Acronym**	**Provider**	**Catalogue #**
BD PrecisionGlide Needle gauge 18 × 1 ½	18G	BD Biosciences, Mississauga, ON, Canada	305196
BD PrecisionGlide Needle gauge 20 × 1 $^{1/3}$	20G	BD Biosciences, Mississauga, ON, Canada	305176
BD PrecisionGlide Needle gauge 22 × 1 $^{1/4}$	22G	BD Biosciences, Mississauga, ON, Canada	305156
Cryopure Tubes, 1 mL	Cryotubes	SARSTEDT, Sarstedtstraße, Nümbrecht, Germany	72.377.992
ECIS Cultureware™ Disposable Electrode Arrays	8W10E+	Applied Biophysics Inc, Troy, NY, United States	8W10E+
Falcon flask 25 cm^2	T25	Corning, Ottawa, ON, Canada	353109
Falcon flask 75 cm^2	T75	Corning, Ottawa, ON, Canada	353136
FALCON Multiwell 6-Well	6-Well plates	Corning, Ottawa, ON, Canada	353046
Ibidi µ-Slide $VI^{0.4}$ Collagen IV	Ibidi Collagen IV	Ibidi, Fitchburg, WI, United Sates	80602
Ibidi µ-Slide $VI^{0.4}$ IbITreat	IbiITeat	Ibidi, Fitchburg, WI, United Sates	80606
Qualitative Grade Plain Filter Paper Sheets	filter paper	Fisher Scientific, Ottawa, ON, Canada	09-802-1B
Sterile syringe 10 mL	syringe	BD Biosciences, Mississauga, ON, Canada	309605
STERITOP-GP 45 mm 500 mL	Filter top	Fisher Scientific, Ottawa, ON, Canada	SCGPT05RE
Thermo Scientific™ Nalgene™ Oak Ridge High-Speed Polycarbonate Centrifuge Tubes	Ultracentrifuge tube	Fisher Scientific, Ottawa, ON, Canada	05-529C
Transwell® Permeable supports<br>6.5 mm Insert, 24 well plate<br>0.4 µm Polyester membrane<br>Thickness 10 µm<br>Tissue Culture Treated, Polystyrene	24 well plate	Corning incorporated, Kennebunk, ME, USA	Costar 3470
