# Supplementary material for: Isolation of endothelial cells, pericytes and astrocytes from mouse brain
Source: PLoS One. 2019 Dec 18;14(12):e0226302. doi: 10.1371/journal.pone.0226302 (PMC6919623; doi:10.1371/journal.pone.0226302)
Supplement: S4 Table — (PDF) [file pone.0226302.s012.pdf]

S4 Table. Detailed list of used equipment

Equipment			
**Item**	**Acronym**	**Provider**	**Catalogue #**
Leica-TCS-SP5	Confocal microscope	Leica Microsystems Inc., Concord, ON, Canada	N.A.
Biometra TProfessional Basic 96 gradient	ThermoCycler	Analytik Jena	N.A.
BD FACSDiva	flow cytometry software	BD Biosciences, Mississauga, ON, Canada	v8.0.1
BD LSR II Flow Cytometry Cell Analyzer	Flow cytometry	BD Biosciences, Mississauga, ON, Canada	N.A.
Bead bath Lab armor	warming bath	Sheldon Manufacturing, Cornelius, OR, United States	74300-714
Corning LSE benchtop shaking incubator	shaking incubator	Corning, Ottawa, ON, Canada	6790
ECIS Zθ and 16W array station	ECIS	Applied Biophysics Inc, Troy, NY, United States	Software v1.2.215.0 PC
Endohm-6 cup	Cup	World Precision Instruments, Sarasota, FL, United States	
Fluorescence elumination system X-cite	fluorescence source	Excelitas, Vaudreuil-Dorion, QC, Canada	120PC Q
Forma™ Steri-Cycle™ CO2 Incubators	incubator	Thermo Fisher Scientific, Burlington, ON, Canada	370
IX81 Inverted Microscope	Fluorescence microscope	Olympus, Waltham, MA, United States	IX81
Leica MZ16 stereo microscope	dissecting microscope	Leica Microsystems Inc., Concord, ON, Canada	N.A.
MetaMorph Advanced	acquisition software	Molecular Devices, Sunnyvale, CA, United States	v7.8.9.0
Motic Image plus 3.0	acquisition software	Moticam, Kowloon Bay, Kowloo, Hong Kong	N.A.
MOTICAM 3+ 3.0 MP	camera	Moticam, Kowloon Bay, Kowloo, Hong Kong	
Mr. Frosty Freezing container	freezing container	Thermo Fisher Scientific, Burlington, ON, Canada	15-350-50
MILLICELL-ERS	TEER	MILLIPOR, Burlington, MA, United States	N.A.
Retiga 2000R CCD Camera	camera	QIMAGING, Surrey, BC, Canada	RET-2000R-F-M-12-C
Sorvall RC 6 Superspeed centrifuge	ultracentrifuge	Thermo Fisher Scientific, Burlington, ON, Canada	501057486-6

Sorvall ST 16R centrifuge	centrifuge	Thermo Fisher Scientific, Burlington, ON, Canada	75004240
Worthington Liquid Nitrogen Refrigerator	azote Tank	Worthington industries, Columbus, OH, United States	XT34
ZEISS Axiovert S100	bright field microscope	Carl Zeiss Microscopy LLC, Peabody, MA, United States	Axiovert S100
