# Supplementary material for: Isolation of endothelial cells, pericytes and astrocytes from mouse brain
Source: PLoS One. 2019 Dec 18;14(12):e0226302. doi: 10.1371/journal.pone.0226302 (PMC6919623; doi:10.1371/journal.pone.0226302)
Supplement: S5 Table — (PDF) [file pone.0226302.s013.pdf]

S5 Table. FACS antibodies and isotypes.

Target	Host/Isotype	Fluorochrome	Provider	Catalogue	Quantity (µg/test)
**PDGFR-β**	Rat/IgG2a, kappa	PE	eBioscience	12-1402-81	0.6
**PECAM-1**	Rat/IgG2a, kappa	PE-Cy7	Biolegend	102418	0.25
**GLAST-1**	Mice/IgG2a	APC	Milteny Biotec	130-098-803	0.5
**GFAP**	Mice/IgG1, kappa	A488	ThermoFisher scientific	A-21294	0.5
**CD45**	Rat/IgG2b, kappa	PerCP-Cy5.5	BDbioscience	550994	0.25
**CD11b**	Rat/IgG2b, kappa	A700	BDbioscience	557960	0.4
