# Supplementary material for: Isolation of endothelial cells, pericytes and astrocytes from mouse brain
Source: PLoS One. 2019 Dec 18;14(12):e0226302. doi: 10.1371/journal.pone.0226302 (PMC6919623; doi:10.1371/journal.pone.0226302)
Supplement: S6 Table — (PDF) [file pone.0226302.s014.pdf]

S6 Table. Confocal and ICC antibodies.

Target	Host/Isotype	Antibody	Fluorophore	Provider	Catalogue	Dilution
**ALCAM**	Rabbit/IgG	Primary	N.A.	Abcam	ab109215	1:1000
**Claudin-5**	Rabbit/IgG	Primary	N.A.	Invitrogen	34-1600	1:200
**Donkey anti Rabbit**	Donkey/IgG	Secondary	A488	Invitrogen	A32790	1:400
**Donkey anti Rat**	Donkey/IgG	Secondary	Cy3	Milipore-Sigma	AP189C	1:200
**GFAP**	Mousse/IgG1k	Primary	A488	Life Technologie	A21294	1:1000
**GLAST-1**	Mousse/IgG2a k	Primary	APC	Miltenyi Biotec	130-098-803	1:500
**Goat anti Rabbit**	Goat/IgG	Secondary	A488	Invitrogen	A-11034	1:500
**JAM-A**	Rat/IgG	Primary	N.A.	Abcam	ab180821	1:200
**MCAM**	Rabbit/IgG	Primary	N.A.	Abcam	Ab75769	1:2000
**NG2**	Mousse/IgG1	Primary	A488	Milipore-Sigma	MAB5384 A4	1:1000
**Occludin**	Mouse / IgG1	Primary	A594	Invitrogen	331594	1:100
**PDGFR-β**	Rabbit/IgG	Primary	N.A.	Invitrogen	MA5-15143	1:400
**PECAM**	Rat/ IgG2a, κ	Primary	N.A.	BDbioscience	558736	1:150
**S100b**	Rabbit/	Primary	A488	Abcam	ab196442	1:100
**VE-Cadh**	Rat/IgG2a, κ	Primary	N.A.	BDbioscience	555289	1:100
**ZO-1**	Rabbit/IgG	Primary	N.A.	Invitrogen	40-2200	1:1000
**ZO-2**	Rabbit/IgG	Primary	N.A.	Invitrogen	71-1400	1:100
**A-SMA**	Mousse/ IgG2a, k	Primary	A488	eBioscience	53-9760-82	1:1000
**FSP-1**	Rabbit/IgG	Primary	A488	Abcam	ab208566	1:100
