# Supplementary material for: Isolation of endothelial cells, pericytes and astrocytes from mouse brain
Source: PLoS One. 2019 Dec 18;14(12):e0226302. doi: 10.1371/journal.pone.0226302 (PMC6919623; doi:10.1371/journal.pone.0226302)
Supplement: S7 Table — (PDF) [file pone.0226302.s015.pdf]

S7 Table. q-PCR probes and primers

Target	Genes	Efficiency	UPL [a]	Sequence reference	Primer A	Primer B
Cldn-5	Cldn5	98.6	52	NM_013805.4	TTAAGGCACGGGTAGCACTC	ATGTTGGCGAACCAGCAG
Ocldn	Ocln	94.1	10	NM_008756.2	GTCCGTGAGGCCTTTTGA	GGTGCATAATGATTGGGTTTG
ZO-1 [b]	ZO-1	94.0	81	NM_009386.2, NM_001163574.1	CGCGGAGAGAGACAAGATGT	GAAGCGTCACTGTGTGCTGT
P-gp	Abcb1a	97.0	18	NM_011076.2	GGGCATTTACTTCAAACTTGTCA	TTTACAAGCTTCATTTCCTAATTCAA
BCRP	Abcg2	97.0	25	NM_011920.3	AGGTCTGGAAAAAGTAGCAGATTC	CTCCATCCCTATGCTTGTCC
GLUT-1	Slc2a1	101	4	NM_011400.3	GTATCCTGTTGCCCTTCTGC	TCGAAGCTTCTTCAGCACAC
Hprt	Hprt	91.2	95	NM_013556.2	TCCTCCTCAGACCGCTTTT	CCTGGTTCATCATCGCTAATC
Gapdh	Gapdh	91.2	80	NM_008084.2	TGTCCGTCGTGGATCTGAC	CCTGCTTCACCACCTTCTTG
Tbp	Tbp	105	97	NM_013684.3	GGGAGAATCATGGACCAGAA	GATGGGAATTCCAGGAGTCA

[a]: Universal Probe Library from Roche Life Science.

[b]: Two primer pairs were used to detect the two isoforms of zonula occludens 1 (ZO-1).
